# Isolation, structural elucidation, and integrated biological and computational evaluation of antidiabetic labdane diterpenes from *Curcuma zedoaria* rhizomes†

**DOI:** 10.1039/d5ra03418c

**Published:** 2025-06-26

**Authors:** Tho Huu Le, Diem Ngoc Thi Lu, Hai Xuan Nguyen, Phu Hoang Dang, Truong Nhat Van Do, Nguyen Thien Han Le, Thang Quoc Truong, Minh Hien Nguyen, Mai Thanh Thi Nguyen

**Affiliations:** a Faculty of Chemistry, University of Science, Vietnam National University Ho Chi Minh City Vietnam nttmai@hcmus.edu.vn; b Vietnam National University Ho Chi Minh City Vietnam nmhien@uhsvnu.edu.vn; c Research Lab for Drug Discovery and Development, University of Science Ho Chi Minh City Vietnam; d University of Health Sciences, Vietnam National University Ho Chi Minh City Vietnam

## Abstract

The phytochemical investigation of the EtOAc-soluble extract of the rhizomes of *Curcuma zedoaria* (Berg.) Roscoe led to the isolation of five labdane-type diterpenes, including a previously undescribed norditerpene, zedolabdin A (CZ1), and four known compounds (CZ2–CZ5). The structures of these compounds were elucidated using NMR, HR-ESI-MS, and IR spectroscopy, supported by comparisons with literature data. The anti-α-glucosidase evaluation revealed that all compounds exhibited potent inhibitory activity, with zerumin (CZ3) and coronarin C (CZ4) displaying the most potent inhibition, achieving IC_50_ values of 6.2 μM and 3.0 μM, respectively, significantly lower than the positive control, acarbose (IC_50_ = 190.6 μM). Molecular docking and dynamics studies identified coronarin C (CZ4) and zedolabdin A (CZ1) as the most promising candidates for α-glucosidase inhibition, exhibiting strong interactions and structural stability. *In silico* ADMET and toxicity predictions indicated that CZ1 and CZ4 had favorable safety and pharmacokinetic profiles, whereas CZ2 and CZ3 posed higher toxicity risks, with CZ3 also showing potential CYP3A4 inhibition. These findings suggest that CZ1 and CZ4 hold significant potential as novel α-glucosidase inhibitors (AGIs), supporting their further development as safe and effective antidiabetic agents. Moreover, the structural features of CZ1, particularly its hydrogen bonding and hydrophobic interactions, contribute to its enhanced binding affinity and stability within the enzyme's active site. Similarly, CZ4's favorable interactions and pharmacokinetic properties reinforce its potential as a promising AGI candidate, warranting further optimization for drug development.

## Abstract 2

The phytochemical investigation of *Curcuma zedoaria* rhizomes revealed that a new norditerpene, zedolabdin A (CZ1), and coronarin C (CZ4) exhibited the most promising α-glucosidase inhibitory activity and favorable ADMET profiles, supporting their potential as safe and effective antidiabetic agents.

## Graphical abstract

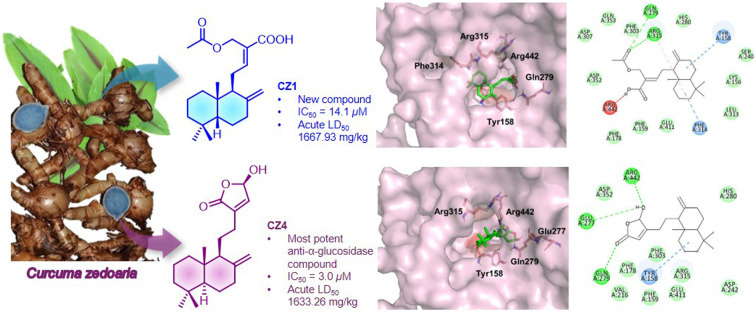

## Introduction

Type 2 diabetes mellitus (T2DM) is a persistent metabolic disorder characterized by insulin resistance and inadequate insulin production, resulting in elevated glucose levels in the bloodstream.^1^ Effective postprandial glucose control is essential for managing T2DM, and α-glucosidase inhibitors (AGIs) are commonly used to address this.^2,3^ These glycosidase enzymes are found on the brush border membrane of the small intestine, responsible for catalysing the hydrolysis of α-glycosidic bonds in carbohydrates from α-amylase-digested starch, breaking them down into monosaccharides for absorption in the intestine.^4^ AGIs function by blocking the α-glucosidase, which delays the digestion and absorption of carbohydrates, thereby reducing rapid glucose spikes after meals.^1^

α-Glucosidases are generally categorized into two main groups, GH-family 13 and 31, based on their sequence similarity. The enzyme's catalytic GH-31 domain (residues 334–779) is conserved, while a variable loop from the *N*-terminal domain (residues 271–288) plays a role in shaping the substrate binding site.^5^ Previous studies have focused on the inhibition of α-glucosidase through the docking of diterpenoids using a homology model constructed from the α-glucosidase sequence. This analysis revealed that the 18,19-γ-lactone forms a water-mediated hydrogen bond with H245, while the diterpene structure interacts with a hydrophobic surface within a five-residue binding pocket.^6^ Despite the availability of several AGIs, many are associated with side effects, highlighting the need for new, safer compounds with enhanced efficacy.

Although *in vitro* assays to evaluate AGIs are well-established and crucial in drug development, the gap between *in vitro* findings and the development of drug candidates remains significant. From preliminary studies to a compound's commercial and clinical use, it often spans over a decade and incurs costs exceeding $1 billion.^7^ Moreover, statistics reveal that a significant proportion of drug candidates fail during development, primarily due to toxicity concerns and, most critically, a lack of clinical efficacy.^8,9^ Current computational tools extend beyond activity prediction to include the assessment of absorption, distribution, metabolism, excretion, and toxicity (ADMET) profiles of compounds. These predictions are instrumental in identifying compounds with favorable pharmacokinetic and safety profiles, reducing the risk of late-stage failures. By integrating virtual screening, a process that uses computer simulations to identify potential drug candidates, with ADMET predictions, researchers can prioritize compounds for subsequent *in vitro* and *in vivo* studies, streamlining the selection process and enhancing the efficiency of drug discovery pipelines.^10,11^ Furthermore, computational tools enable to predict natural compound interactions with drug targets, providing valuable insights into how specific functional groups or structural motifs contribute to biological activity.^12,13^

*Curcuma zedoaria* (Berg.) Roscoe, commonly known as “Nghệ đen” in Vietnam, is a member of the Zingiberaceae family and has a long-standing history of medicinal use. The rhizomes of *C. zedoaria* are traditionally used in folk remedies to treat gastrointestinal issues, including bloating, indigestion, and gastritis.^14,15^ This plant has gained recognition for its bioactive compounds, particularly sesquiterpenoids and diterpenoids.^16–19^ Among the diterpenoids, labdane-type compounds are noteworthy for their broad pharmacological activities, including anti-inflammatory,^20^ antimicrobial,^21^ and cytotoxic effects.^22^ However, their potential as AGIs has not been fully explored. Given the therapeutic importance of AGIs in managing postprandial glucose levels, investigating labdane-type diterpenoids from *C. zedoaria* could lead to the identification of novel bioactive compounds for the development of new antidiabetic therapies.

In this study, five labdane-type diterpenoids, including a new compound, were isolated from the rhizomes of *C. zedoaria*. These compounds were evaluated for α-glucosidase inhibitory activity, and all isolates exhibited potent inhibition. The promising results suggest that these compounds have potential as AGIs, offering new possibilities for diabetes management. Although further studies, including molecular docking, and *in silico* toxicity predictions, are necessary to understand their mechanisms and safety profiles fully, this research serves as a crucial step towards the development of labdane-type diterpenoids as antidiabetic agents. By isolating and characterizing bioactive diterpenoids, this study bridges the gap between virtual screening predictions and experimental validation, providing a comprehensive approach to exploring the therapeutic potential of natural products in diabetes management.

## Results and discussion

**Fig. 1:**
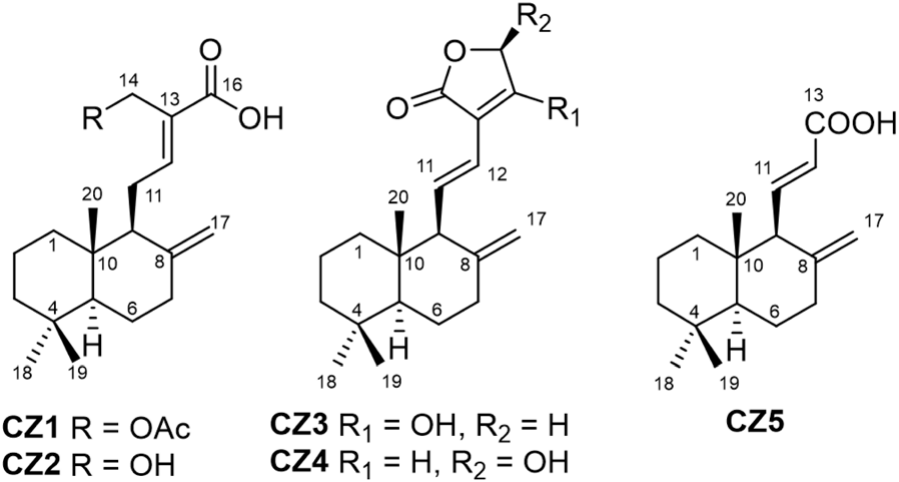
Chemical structures of isolated compounds (CZ1–CZ5) from *C. zedoaria*.

Five diterpenes (CZ1–CZ5) were isolated from the EtOAc extract of *C. zedoaria* rhizomes using a combination of column chromatography and preparative thin-layer chromatography, employing various solvent systems for elution. Their structures were charaterized by using spectroscopic techniques, consisting of a novel compound, zedolabdin A (CZ1), along with four known compounds, namely (*E*)-14-hydroxy-15-norlabda-8(17),12-dien-16-oic acid (CZ2),^23^ zerumin (CZ3),^24^ coronarin C (CZ4)^25^ and (*E*)-14,15,16-trinorlabda-8(17),11-dien-13-oic acid (CZ5)^26^ (Fig. 1).

Compound CZ1 was obtained as a colorless gel. The HR-ESI-MS spectrum revealed *m*/*z* 349.23775 for the protonated molecular ion [M + H]^+^ (calcd for 349.23734), indicating its molecular formula C_21_H_32_O_4_. The IR spectrum exhibited absorption for a double bond (3084 and 1715 cm^−1^), carboxylic acid (2924 and 1698 cm^−1^), and ester (1735 cm^−1^). The ^1^H-NMR spectrum of CZ1 revealed three olefinic protons [*δ*_H_ 7.12 (1H, t, *J* = 6.5 Hz, H12), 4.83 (1H, brs, H17b) and 4.38 (1H, brs, H17a)], one oxymethylene group [*δ*_H_ 4.86 (2H, s, H14)], two methine groups [*δ*_H_ 1.87 (1H, d, *J* = 10.4 Hz, H9), 1.12 (1H, dd, *J* = 12.6 and 3.0 Hz, H5)], six methylene groups [*δ*_H_ 1.07–2.55] along with three methyl groups [*δ*_H_ 0.88 (3H, s, H18), 0.82 (3H, s, H19) and 0.73 (3H, s, H20)] and one acetyl group [*δ*_H_ 2.06 (3H, s, 14–OCOCH_3_)]. The ^13^C NMR spectrum exhibited resonances of 21 carbons, including one carboxylic carbon [*δ*_C_ 170.7 (C16)], one carbonyl carbon of an acetoxyl group [*δ*_C_ 170.8 (14–OCOCH_3_)], four olefinic carbons [*δ*_C_ 153.7 (C12), 148.1 (C8), 125.6 (C13), 107.7 (C17)]. In the high field exhibited signals of one oxymethylene carbon [*δ*_C_ 58.0 (C14)], two quaternary sp^3^ carbons [*δ*_C_ 39.5 (C10) and 33.7 (C4)], two methines [*δ*_C_ 56.5 (C9) and 55.3 (C5)], six methylenes [*δ*_H_ 19.2–41.9], three methyl carbons [*δ*_C_ 33.7 (C18), 21.9 (C19), and 14.6 (C20)] together with an acetyl group [*δ*_C_ 20.7 (14–OCOCH_3_)] (Table 1).

**Table 1:** 1D-NMR data of compound CZ1 measured in CDCl_3_

Position	*δ*_H_ mult. (*J* in Hz)	*δ*_C_
1	1.71 m	39.1 CH_2_
	1.07 ddd (12.9, 12.8, 3.9)	
2	1.58 ddddd (13.8, 13.4, 12.8, 4.1, 4.0)	19.2 CH_2_
	1.51 m	
3	1.41 br d (13.3)	41.9 CH_2_
	1.18 ddd (13.4, 13.3, 4.2)	
4	—	33.5 qC
5	1.12 dd (12.6, 3.0)	55.3 CH
6	1.74 m	24.2 CH_2_
	1.34 dddd (12.9, 4.4, 4.4, 3.0)	
7	2.00 ddd (12.9, 5.1, 4.4)	37.8 CH_2_
	2.40 m	
8	—	148.1 qC
9	1.87 d (10.4)	56.5 CH
10	—	39.5 qC
11	2.55 ddd (13.5, 10.4, 6.5)	24.0 CH_2_
	2.40 m	
12	7.12 t (6.5)	153.7 CH
13	—	125.6 qC
14	—	170.7 qC
15	—	—
16	4.86 s	58.0 CH_2_
17	4.38 brs	107.7 CH_2_
	4.83 brs	
18	0.88 s	33.5 CH_3_
19	0.82 s	21.6 CH_3_
20	0.73 s	14.3 CH_3_
14–OCOCH_3_	2.06 (3H, s)	170.8 qC, 20.7 CH_3_

**Fig. 2:**
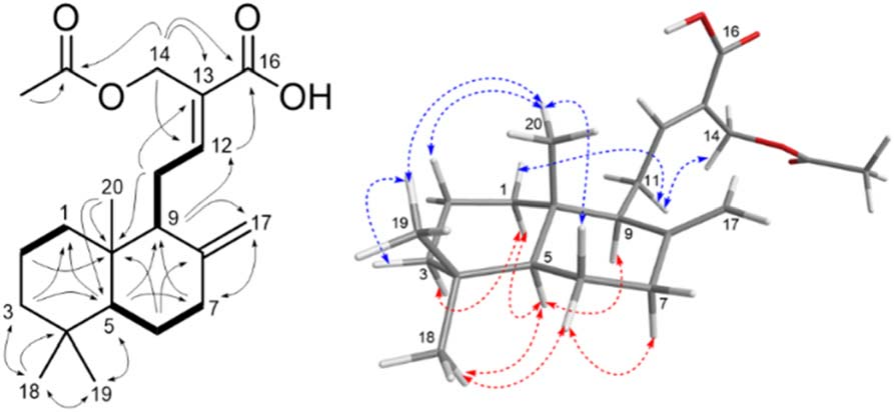
The HMBC (solid arrows), COSY (bond lines), and NOESY (dashed arrows) correlations for zedolabdin A (CZ1).

Combining the 1D-2D NMR data, compound CZ1 exhibits the structure of a norditerpenoid labdane-type skeleton. This structure was clarified by HMBC and COSY correlations (Fig. 2). The acid carboxylic group attached at the C13 was confirmed through HMBC correlations of protons H14/H12 with C16 and the chemical shift at [*δ*_C_ 170.7 (C16)]. The acetoxyl group was identified at the C14 through the HMBC correlations from oxymethylene protons H14 to the carbonyl carbon of the acetoxyl group, together with the chemical shift at [*δ*_C_ 170.8 (14–OCOCH_3_)]. The presence of double bonds at C12=C13 was deduced from HMBC correlations of H12 to C9/C16, as well as H9/H14 to C12, supported by the chemical shift of olefinic carbon C12 (*δ*_C_ 153.7 ppm) and H11/H16 with C13, alongside the chemical shift at C13 (*δ*_C_ 125.6 ppm). Similarly, the HMBC interactions from H17 to C7/C9, and H9/H7 to C17, with the carbon signals at C17 (*δ*_C_ 107.7 ppm), together with the correlations between H6/H11 and C8, supported by the chemical shift of the C8 (*δ*_C_ 148.1 ppm) were confirmed the double bonds at C8 and C17 (Fig. 2). The 1D and 2D NMR spectra of compound CZ1 exhibited a pattern comparable to that of compound CZ2, differing only in the substitution at carbon C16, where a hydroxyl group in CZ2 is replaced by an acetoxyl group in CZ1.

**Fig. 3:**
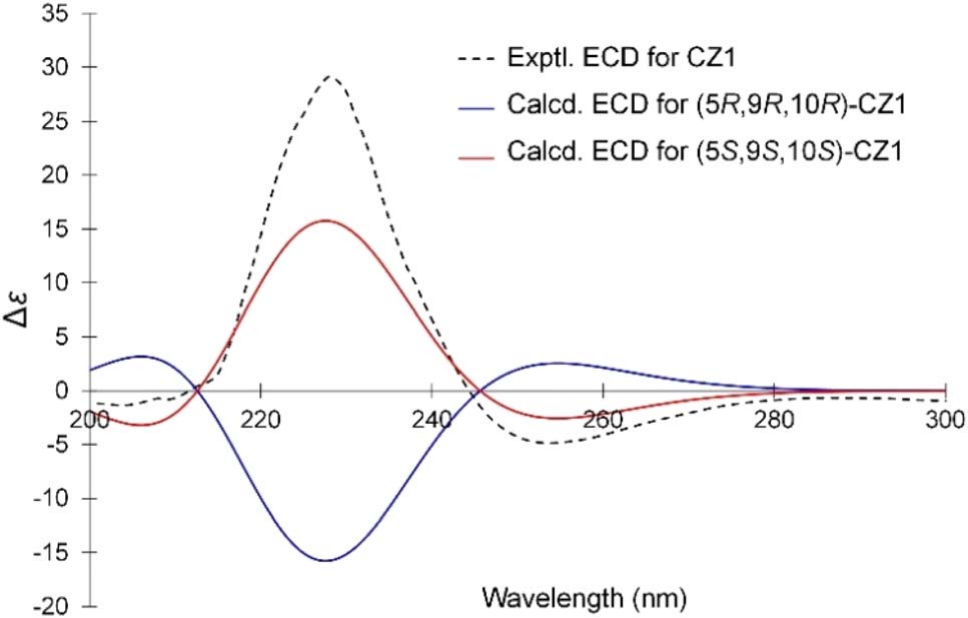
Calculated ECD spectra of (5S,9S,10S)– and (5R,9R,10R)-CZ1 and the experimental ECD spectrum of CZ1 in methanol.

The relative stereochemical structure of CZ1 was elucidated by analyzing the NOESY spectrum. The NOESY correlations from H7 (*δ*_H_ 2.40)/H6 (*δ*_H_ 1.34), H6 (*δ*_H_ 1.34)/H_3_18, and H5/H9 indicated protons H5, H9, H6 (*δ*_H_ 1.34), H7 (*δ*_H_ 2.40), and methyl groups (C(18)H_3_) located on the same plane of the decalin moiety. Meanwhile, the NOESY interactions of H1 (1.71)/H11, H2 (1.58)/H_3_20, H_3_20/H_3_19, H_3_19/H3 (1.41) and H_3_20/H6 (1.74) assigned that protons H1 (1.71), H2 (1.58), H3 (1.41), H6 (1.74), H11 together with two methyl groups (C(19)H_3_ and C(20)H_3_) oriented on the same plane of the decalin moiety. In addition, the NOESY correlations observed between protons H11 and H_2_14, indicated an *E*-configuration for the double bond C12 and C13 (Fig. 2). The NOESY experiment confirmed its relative stereochemistry of the skeleton. Based on a comparison of experimental and calculated electronic circular dichroism (ECD) data, the absolute configuration of compound CZ1 was determined. The process began with initial conformational searches utilizing the MMFF force field. The resulting structures were subsequently re-optimized at the B3LYP/6-31G* level using Spartan'14. All conformers contributed over 95% Boltzmann distribution were selected for subsequent DFT (Density Functional Theory) calculations in Gaussian 16. TDDFT (Time-Dependent Density Functional Theory) calculations were then carried out at the B3LYP/6-31G*/CAM-B3LYP/aug-cc-pVDZ level to generate Boltzmann-weighted ECD spectra. The calculated ECD spectrum for the (5S,9S,10S)-CZ1 showed a close match with the experimental spectrum (Fig. 3). All the aforementioned evidence facilitated the elucidation of the structure of CZ1, which is named zedolabdin A.

Five isolated compounds were examined to assess their inhibitory effect on α-glucosidase at concentrations ranging from 1 to 100 μM. The concentration of these compounds for the 50% inhibition of α-glucosidase (IC_50_) is displayed in Table 2. This study used acarbose, a pharmacological α-glucosidase inhibitor in managing hyperglycaemia, as a positive control (PC).^27^

**Table 2:** α-Glucosidase inhibitory activity of five isolated compounds (CZ1–CZ5) from *C. zedoaria* rhizomes

Compounds	Inhibition rate (%)				IC_50_ (μM)
	100 μM	50 μM	25 μM	10 μM	
CZ1	98.8 ± 0.4	96.2 ± 0.4	81.4 ± 0.5	29.5 ± 1.8	14.1
CZ2	99.1 ± 2.0	72.4 ± 2.0	46.3 ± 3.1	24.6 ± 4.1	25.5
CZ5	99.8 ± 0.2	75.8 ± 4.4	16.6 ± 3.8	3.5 ± 3.6	37.6
	**10 μM**	**5 μM**	**2.5 μM**	**1.0 μM**	
CZ3	73.4 ± 2.3	35.2 ± 1.9	20.8 ± 3.6	6.9 ± 4.0	6.2
CZ4	95.4 ± 1.5	81.0 ± 1.7	34.0 ± 0.9	13.7 ± 5.4	3.0
	**250 μM**	**100 μM**	**50 μM**	**25 μM**	
PC	66.0 ± 1.2	32.1 ± 1.7	19.7 ± 1.3	8.6 ± 1.1	190.6

The results indicated that all five compounds (CZ1–CZ5) exhibited remarkable α-glucosidase inhibitory activity, with IC_50_ values ranging from 3.0 to 37.6 μM, surpassing the potency of the positive control. Among them, zerumin (CZ3) and coronarin C (CZ4) demonstrated exceptional inhibitory effects, with IC_50_ values of 6.2 μM and 3.0 μM, respectively. These findings suggest that the lactone ring closure at the C16 position significantly enhances the structural potency of these compounds, consistent with previous reports in the literature.^28,29^ Moreover, acetylation at the C14 position was observed to double the enzyme inhibitory activity. This was evident from the bioactivity results of compound CZ1 (IC_50_ = 14.1 μM) and compound CZ2 (IC_50_ = 25.5 μM), further emphasizing the critical influence of specific structural modifications on α-glucosidase inhibitory potential.

Although regarding *in vitro* enzyme assay, the *Saccharomyces cerevisiae* α-glucosidase enzyme (EC 3.2.1.20) was used due to its commercial availability, its 3D crystallographic structure is not available in public databases, the *in silico* study was processed on *S. cerevisiae* isomaltase structure (PDB: 3A4A) with high sequence similarity (84%) compared to *S. cerevisiae* maltase structure.^30^ Moreover, both α-glucosidase and isomaltase hydrolyze sucrose and the synthetic substrate *p*-nitrophenyl-α-d-glucopyranoside.^31^ These reasons suggest that isomaltase is a potential alternative model for computational analysis, allowing for the primary investigation of enzyme–ligand interactions.

**Fig. 4:**
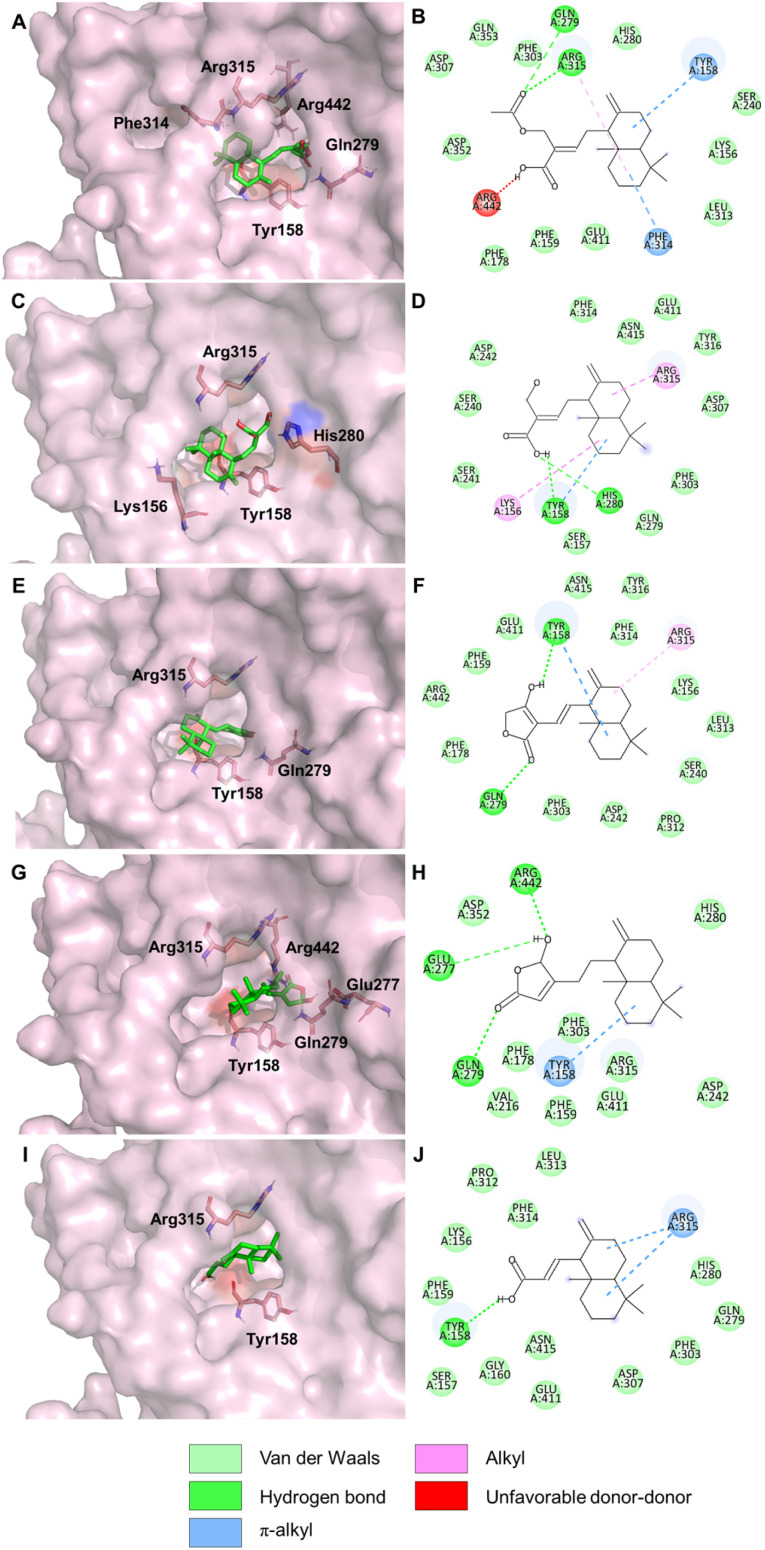
3D docking poses and 2D interactions diagrams of α-glucosidase binding compounds: CZ1 (A, B); CZ2 (C, D); CZ3 (E, F); CZ4 (G, H) and CZ5 (I, J).

The binding interactions of five compounds from *C. zedoaria* with α-glucosidase are summarized in Table 3 and Fig. 4. Three compounds, zerumin (CZ3), coronarin C (CZ4), and zedolabdin A (CZ1), demonstrated the highest binding scores, with binding energies of −8.8, −8.7, and −8.4 kcal mol^−1^, respectively. In contrast, (*E*)-14-hydroxy-15-norlabda-8(17),12-dien-16-oic acid (CZ2) and (*E*)-14,15,16-trinorlabda-8(17),11-dien-13-oic acid (CZ5) showed lower binding energies of −7.6 and −7.8 kcal mol^−1^. These computational results align well with the *in vitro* assay findings (Table 2), where compounds CZ3 and CZ4 exhibited the most potent α-glucosidase inhibitory activity, followed by CZ1, CZ2, and CZ5. Notably, all five compounds share a decaline moiety, which plays a crucial role in binding affinity by forming π-alkyl interaction with the Tyr158 residue of α-glucosidase and blocking the entrance active site of α-glucosidase. This aligned with previous studies that identified these compounds as competitive inhibitors binding near the active site of α-glucosidase.^32,33^ Additionally, Arg315 showed a role in stabilizing the enzyme–compound complex through various interactions, further enhancing binding affinity. On the other hand, the lactone moiety of compounds CZ3 and CZ4 interacted with Glu277 and Gln279 (Fig. 4E and G), effectively blocking the enzyme's exit site. This obstruction inhibited the enzyme product's release, impeding the catalytic activity of α-glucosidase. The difference in the position of hydroxyl substituents on the furanone moiety significantly influenced the binding affinity with the enzyme. This observation is consistent with the binding energy scores and *in vitro* findings, where compound CZ4 demonstrated the lowest IC_50_ value, followed by compound CZ3, indicating their superior inhibitory activity. To sum up, this finding suggests that compounds CZ3 and CZ4 may inhibit α-glucosidase through a competitive or uncompetitive mechanism, potentially interfering with substrate entrance or releasing exit. Within the scope of the current study, molecular docking provided preliminary insights into the potential mode of inhibition, suggesting either competitive or uncompetitive binding. However, future studies should incorporate enzyme kinetic assays to experimentally validate computational predictions and gain a more comprehensive understanding of the inhibitor's mechanism of action.

**Table 3:** Molecular docking interaction of five compounds (CZ1–CZ5) from *C. zedoaria* and α-glucosidase

Cp	Binding energy (kcal mol^−1^)	Residue interacts
CZ1	−8.4	Hbond: Gln279 (2.6 Å)
		π-alkyl/alkyl: Tyr158 (3.6 Å), Phe314 (3.4 Å), Arg315 (3.5 Å)
		Unfavorable bond: Arg442 (4.4 Å)
CZ2	−7.6	Hbond: Tyr158 (3.7 Å), His280 (2.4 Å)
		Alkyl: Arg315 (3.9 Å), Lys156 (3.4 Å)
		π-alkyl: Tyr158 (4.0 Å)
CZ3	−8.8	H bond: Tyr158 (2.4 Å), Gln279 (2.4 Å)
		π-alkyl/alkyl: Tyr158 (4 Å), Arg315 (3.8 Å)
CZ4	−8.7	H bond: Glu277 (2.0 Å), Gln279 (2.5 Å), Arg442 (2.2 Å)
		π-alkyl: Tyr158 (3.8 Å)
CZ5	−7.8	H bond: Tyr158 (1.9 Å)
		Alkyl: Arg315 (3.7 Å)

**Fig. 5:**
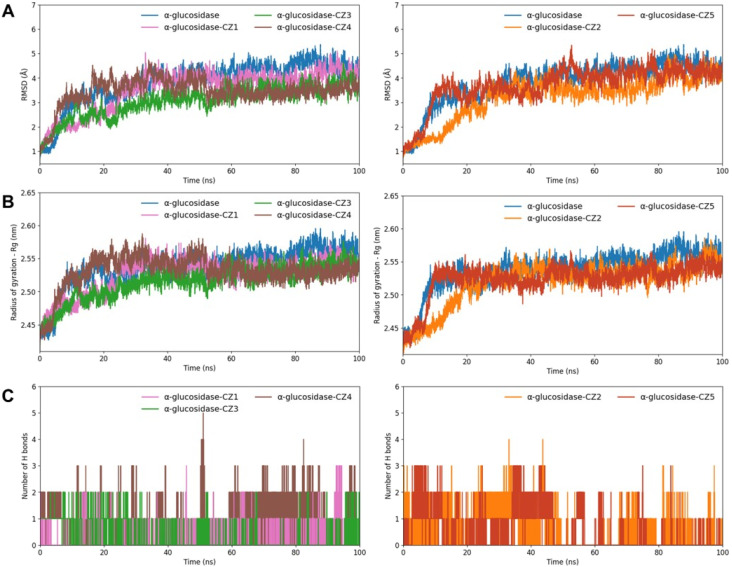
MD simulation of RMSD (A), radius of gyration (B) and number of hydrogen bonds (C) over times of α-glucosidase with zedolabdin A (CZ1; pink), zerumin (CZ3; green), coronarin C (CZ4; brown), (*E*)-14-hydroxy-15-norlabda-8(17),12-dien-16-oic acid (CZ2; orange), compound (CZ5; red) and without binding compounds (blue).

A molecular dynamics simulation assessed the stability, flexibility, and interactions between α-glucosidase and the five compounds during 100 ns, as shown in Fig. 5. The initial 50 ns allowed the complexes to achieve a stable stage. Among the six simulations, the backbone root-mean-square deviation (RMSD) values for the complexes of α-glucosidase with zerumin (CZ3) and coronarin C (CZ4) were the lowest, stabilizing around 3 Å, which was lower than the unbound α-glucosidase and the complexes with compounds CZ2 and CZ5 (Fig. 5A). Conversely, the RMSD values for the complexes with compounds CZ2 and CZ5 were higher, indicating greater flexibility and less stability. Overall, the RMSD results revealed that α-glucosidase–compound complexes had lower RMSD values compared to the unbound enzyme, aligning with decreased radius of gyration values (Fig. 5B). These findings suggest that the complexes adopt more rigid and stable conformations than the unbound α-glucosidase. Moreover, the MD simulation further revealed that CZ4 formed the highest number of hydrogen bonds among all five compounds, highlighting its robust binding interaction and stability within the complex.

These molecular dynamics results are consistent with molecular docking and *in vitro* assay results, which demonstrated that coronarin C (CZ4) exhibits promising α-glucosidase inhibitory potential. The MD simulation further revealed the flexibility across all amino acids *via* root-mean-square fluctuation (RMSF) (Fig. 5D). Residues located in the B domain loop (amino acids 130–160), the active site lid (amino acids 230–236), and the A domain side (amino acids 310–316) exhibited greater fluctuations, corresponding to the substrate-binding pocket formed at the interface of domains A and B of the enzyme.^34^ Notably, the α-glucosidase–CZ4 complex exhibited lower fluctuations compared to α-glucosidase and other enzyme-compound complexes (Fig. 5D, brown line). The reduced RMSF values indicate that CZ4 stabilized the enzyme structure by limiting residue fluctuations through strong interactions. This observation aligns with the molecular docking results (Fig. 4G and H), confirming the stability of the α-glucosidase–CZ4 complex.

The comprehensive results of the *in silico* ADMET prediction of five compounds from SwissADME web tool were demonstrated in Table 4. Importantly, all five compounds adhere to Lipinski's Rule of Five, suggesting they are likely to be bioavailable orally. The findings also indicate a high probability of gastrointestinal absorption for these compounds, contributing to their favourable bioavailability.

**Table 4:** *In silico* ADME profiles of five compounds (CZ1–CZ5) from *C. zedoaria*

Compounds	CZ1	CZ2	CZ3	CZ4	CZ5
Molecular weight	348.48	306.44	316.43	318.45	262.39
No. H-bond acceptor	4	3	3	3	2
No. H-bond donor	1	2	1	1	1
No. Rotatable bonds	6	4	2	3	2
TPSA (Å^2^)	63.6	57.53	46.53	46.53	37.3
Log *P*	3.11	2.72	3.39	3.24	2.85
Log *S*	−4.63	−4.14	−4.69	−4.81	−4.37
Gastrointestinal absorption	High	High	High	High	High
Log kp (cm s^−1^)	−5.02	−5.17	−4.82	−4.64	−4.49
CYP1A2 inhibitor	No	No	No	No	No
CYP2C19 inhibitor	Yes	Yes	Yes	Yes	Yes
CYP2C9 inhibitor	Yes	Yes	Yes	Yes	Yes
CYP2D6 inhibitor	No	No	No	No	No
CYP3A4 inhibitor	No	No	Yes	No	No
Lipinski violations	0 violation	0 violation	0 violation	0 violation	0 violation

Cytochrome P450 (CYP) is a class of enzymes predominantly found in the liver and intestines, responsible for metabolizing most drugs through oxidation processes. Compounds that inhibit these CYP enzymes can lead to drug interactions, which may reduce drug efficacy or even cause toxicity. Noteworthy, all five compounds likely inhibit CYP2C9 and CYP2C19. All five compounds tested do not inhibit CYP1A2 and CYP2D6, two critical metabolizing enzymes in the liver. However, compounds CZ1 and CZ3 have a potential probability of inhibiting CYP3A4, while compounds CZ2, CZ4, and CZ5 do not inhibit this enzyme. Therefore, it is essential to consider clinically significant pharmacokinetic interactions for compounds CZ1 and CZ3 when they are coadministered with other drugs, as mechanism-based inhibition of CYP3A4 can lead to pharmacokinetic-pharmacodynamic drug–drug interactions. In future studies, it will be important to assess the *in vivo* pharmacokinetic profiles of all compounds in the presence of CYP3A4 substrates to evaluate interaction risks and ensure safe therapeutic use.

The computational tool Deep-PK was used to assess the predicted toxicity profiles of the compounds. Compounds CZ1, CZ2, CZ4, and CZ5 exhibited mild toxicity, with LD_50_ values in a rat model of 1667.93 and 1889.56, 1633.26, and 1068.93 mg kg^−1^, respectively. These values suggest that these compounds have a relatively low risk of acute toxicity. In contrast, compounds CZ3 displayed significant toxicity, with LD_50_ values falling below 1000 mg kg^−1^, highlighting the need for caution in their potential applications (Table 5). Moreover, the prediction model identified a specific concern regarding compound CZ3, as it demonstrated a potential to bind to the glucocorticoid receptor. This interaction could contribute to adverse clinical outcomes by disrupting normal glucocorticoid signaling pathways. Since the prediction models for each endpoint and values are established based on different model type, training dataset, there may have discrepant results.^35^ Thus, although CZ3 is predicted to be safe in terms of liver toxicity and non-carcinogenicity, its LD_50_ value of 302.2 mg kg^−1^ indicates a relatively high level of acute toxicity. Despite this, none of the compounds showed significant interactions with key receptors such as the androgen receptor, estrogen receptor, thyroid receptor, or hERG channels, often associated with hormonal imbalances and cardiac arrhythmias. Additionally, the compounds were predicted to be safety concerns to carcinogenicity, liver injury, or micronucleus formation. The *in silico* toxicity prediction results above were among the most commonly investigated in toxicology.^36^ These *in silico* predictions suggest that while compounds CZ1, CZ2, CZ4, and CZ5 may hold promise for further research, the potential risks associated with compound CZ3 require careful consideration and further investigation. Although *in silico* techniques cannot fully replace *in vitro* and *in vivo* methods, they provide valuable insights into mechanism-based toxicity for subsequent *in vitro* and *in vivo* experimental validation in establishing toxicity profiles. Numerous computational models based on chemical analogs have been designed for toxicity assessment.^36^ The *in silico* approach in toxicity studies allows researchers to identify potential adverse effects early in the development process, ultimately reducing time and resources.

**Table 5:** *In silico* toxicity profiles of five compounds (CZ1–CZ5) from *C. zedoaria*

Compounds	CZ1	CZ2	CZ3	CZ4	CZ5
Acute LD_50_ (mg kg^−1^)	1667.93	1889.56	302.20	1633.26	1068.93
Carcinogenesis	Safe	Safe	Safe	Safe	Safe
Liver injury	Safe	Safe	Safe	Safe	Safe
Micronucleus	Safe	Safe	Safe	Safe	Safe
hERG inhibitors	Safe	Safe	Safe	Safe	Safe
Androgen receptor	Safe	Safe	Safe	Safe	Safe
Estrogen receptor	Safe	Safe	Safe	Safe	Safe
Glucocorticoid receptor	Safe	Safe	Toxic	Safe	Safe
Thyroid receptor	Safe	Safe	Safe	Safe	Safe

## Conclusion

In this study, five labdane-type diterpenes, including a novel compound named zedolabdin A, were successfully isolated from the rhizomes of *C. zedoaria*, a medicinal plant widely used in Vietnam. The bioactivity testing revealed that all the isolated diterpenoids exhibited superior α-glucosidase inhibitory activity compared to the positive control, acarbose. These promising results contribute valuable insights into the chemical composition of *C. zedoaria* rhizomes and further support the potential of labdane-type diterpenoids as therapeutic agents for the management of T2DM. Molecular docking studies revealed favorable binding interactions with key residues of α-glucosidase, while *in silico* toxicity predictions indicated a low risk of adverse effects. Our findings suggest that these compounds, especially zedolabdin A (CZ1) and coronarin C (CZ4) could be developed as novel AGIs, offering a new avenue for antidiabetic drug discovery.

## Experimental

### General experimental procedures

Optical values were conducted on a Shimadzu UV-1800 spectrophotometer (Shimadzu Pte., Ltd, Singapore). IR spectra were measured with a Shimadzu IR-408 infrared spectrometer (Shimadzu Pte., Ltd, Singapore). NMR spectra were acquired on a Bruker Avance III 500 spectrometer Bruker BioSpin AG, Bangkok, (Thailand), with chemical shifts (*δ*) referenced to deuterated solvents. HR-ESI-MS data were obtained on Bruker micrOTOFQII mass spectrometer (Bruker Singapore Pte., Ltd, Singapore). Column chromatography utilized silica gel 60 (0.06–0.2 mm; Scharlau, Barcelona, Spain) and LiChroprep RP-18 (40–63 μm; Merck KGaA, Darmstadt, Germany). Thin-layer chromatography (TLC) employed Kieselgel 60 F_254_ or RP-18 F_254_ plates from Merck KGaA (Darmstadt, Germany). α-Glucosidase (EC 3.2.1.20), derived from *Saccharomyces cerevisiae* (750 UN), along with *p*-nitrophenyl-α-d-glucopyranoside, was procured from Sigma Chemical Co. (St. Louis, MO, USA). Acarbose and dimethyl sulfoxide were sourced from Merck (Darmstadt, Germany). All other chemicals and solvents were of the highest purity or analytical grade.

### Plant material

The fresh rhizomes of *C. zedoaria* were collected in Tinh Bien district, An Giang province, Vietnam in September of 2017. All 40 kg of fresh rhizomes of *C. zedoaria* were dried and ground to obtain 6.7 kg of dried rhizomes. The sample was identified by MSc Hoang Viet, Faculty of Biology and Biotechnology, University of Science, Ho Chi Minh City, Vietnam. A voucher specimen (MCE0052) has been archived at the Department of Medicinal Chemistry, Faculty of Chemistry, University of Science, Ho Chi Minh City, Vietnam.

### Extraction and isolation

The dried rhizomes of *C. zedoaria* (6.7 kg) were extracted by a Soxhlet extractor, yielding *n*-hexane- (270.0 g), EtOAc- (238.6 g). The EtOAc extract (238.6 g) was roughly separated on a silica gel column chromatography, eluted initially with a gradient solvent of CHCl_3_–MeOH (v/v, 100 : 0 → 20 : 80) to obtain 26 fractions (fractions A–Z).

Fraction M (6.8 g) was chromatographed on silica gel to give 10 subfractions (subfractions M1–M10). Subfraction M4 (1.6 g) was loaded to silica gel column, eluting with a gradient of *n*-hexane–EtOAc (v/v, 100 : 0 → 0 : 100) to afford four subfractions, including M4.1 (70 mg), M4.2 (238 mg), M4.3 (1.0 g) and M4.4 (11 mg). Subfraction M4.1 (70 mg) was further chromatographed on silica gel *via n*-hexane–chloroform gradient mixtures (v/v, 100 : 0 → 20 : 80) to yield the compound CZ5 (5.9 mg). Subfraction M4.2 (238 mg) was fractionated by a silica gel column chromatography employing *n*-hexane–EtOAc mixtures in ascending order of polarity (v/v, 100 : 0 → 40 : 60), producing three subfractions of M4.2.1 (54 mg), M4.2.2 (72 mg), and M4.2.3 (14 mg). 72 mg of subfraction M4.2.2 was further chromatographed on silica gel with a gradient of a *n*-hexane–EtOAc system (v/v, 100 : 0 → 50 : 50) followed by purified over preparative TLC with *n*-hexane–EtOAc–*i*-PrOH mixtures (v/v/v, 80 : 18 : 2) to furnish the compound CZ3 (8.6 mg). Silica gel column chromatography was utilized for further isolation from fraction M5 (1.7 g) using a gradient *n*-hexane–EtOAc mixtures (v/v, 100 : 0 → 0 : 100) as eluents, this process resulted in four subfractions, consisting of M5.1 (86 mg), M5.2 (437 mg), M5.3 (266 mg), and M5.4 (457 mg). Subfraction M5.3 (266 mg) was subjected to silica gel column chromatography and eluted with *n*-hexane–EtOAc gradient mixtures (v/v, 100 : 0 → 30 : 70) to afford three fractions: M5.3.1 (17 mg), M5.3.2 (92 mg), and M5.3.3 (94 mg). Subfraction M5.3.3 (94 mg) was submitted to reversed-phase silica gel column chromatography, eluting with an acetone–H_2_O gradient solvent system (v/v, 10 : 90 → 50 : 50). Thereafter, this fraction was purified by normal-phase preparative TLC with a CH_2_Cl_2_–CHCl_3_–EtOAc–*i*-PrOH (50 : 30 : 18 : 2) mixture to yield the compound CZ4 (3.5 mg). Fraction M7 (830 mg) was fractionated over normal-phase silica gel column chromatography, eluted with a gradient solvent of *n*-hexane–EtOAc (v/v, 100 : 0 → 20 : 80) to divide four subfractions: M7.1 (374 mg), M7.2 (76 mg), M7.3 (191 mg), and M7.4 (71 mg). Subfraction M7.1 (374 mg) was passed over a silica gel column eluted with *n*-hexane–EtOAc mixtures (v/v, 100 : 0 → 20 : 80) to yield three subfractions: M7.1.1 (184 mg), M7.1.2 (56 mg), and M7.1.3 (95 mg). The compound CZ2 (21.7 mg) was obtained through column chromatography on silica gel of subfraction M7.1.1 (184 mg), and consecutively eluted using an isocratic solvent system of *n*-hexane–EtOAc (v/v, 80 : 20). Finally, the compound CZ1 (5.0 mg) was isolated from M7.4 (71 mg) by chromatography on reverse-phase silica gel, using gradient acetone–H_2_O mixtures (v/v, 10 : 90 → 40 : 60) as the eluent.

### Zedolabdin A (CZ1)

Colorless gel; ^1^H and ^13^C-NMR (CDCl_3_, 500 MHz, see Table 1); IR *ν*_max_ (KBr) 3084, 2924, 1735, 1715, 1698 cm^−1^; HR-ESI-MS (*m*/*z*): [M + H]^+^ calcd for C_21_H_33_O_4_, 349.2373; found, 349.2377.

### (*E*)-14-hydroxy-15-norlabda-8(17),12-dien-16-oic acid (CZ2)

Colorless gel; ^1^H NMR (CDCl_3_, 500 MHz) *δ* (ppm) 6.96 (1H, t, *J* = 6.8 Hz), 4.83 (1H, brs), 4.40 (1H, brs), 4.36 (2H, s), 2.50 (1H, ddd, *J* = 13.5, 10.4 and 6.6 Hz), 2.34 (1H, m), 2.35 (1H, m), 2.00 (1H, ddd, *J* = 13.2, 5.3 and 4.3 Hz), 1.85 (1H, d, *J* = 10.4 Hz), 1.72 (2H, m), 1.57 (1H, ddddd, *J* = 13.8, 13.3, 12.9, 4.0 and 4.0 Hz), 1.40 (1H, br d, *J* = 13.4 Hz), 1.32 (1H, dddd, *J* = 12.9, 4.4, 4.3 and 2.8 Hz), 1.18 (1H, dddd, *J* = 13.4, 13.3 and 3.8 Hz), 1.12 (1H, dd, *J* = 12.8 and 2.8 Hz), 1.08 (1H, ddd, *J* = 12.9, 12.9 and 4.0 Hz), 0.88 (3H, s), 0.81 (3H, s, H-19), 0.73 (3H, s); ^13^C NMR (CDCl_3_, 125 MHz) *δ* (ppm) 172.5, 150.3, 148.2, 129.7, 108.0, 57.3, 56.8, 55.5, 42.2, 39.8, 39.4, 38.0, 33.7 (2C), 24.3, 24.0, 21.9, 19.5, 14.6; HR-ESI-MS *m*/*z* [M–H]^−^ 305.2120 (calcd for C_19_H_29_O_3_ 305.2122).

### Zerumin (CZ3)

Colorless gel; ^1^H NMR (CDCl_3_, 500 MHz) *δ* (ppm) 6.33 (1H, d, *J* = 16.2 Hz), 6.00 (1H, dd, *J* = 16.2 and 10.7 Hz), 4.87 (2H, d, *J* = 15.3 Hz), 4.77 (1H, d, *J* = 1.8 Hz), 4.41 (1H, d, *J* = 1.8 Hz), 2.46 (1H, d, *J* = 10.7 Hz), 2.45 (1H, ddd, *J* = 13.6, 4.5 and 2.5 Hz), 2.10 (1H, ddd, *J* = 13.6, 13.3 and 5.6 Hz), 1.72 (1H, dddd, *J* = 12.7, 5.6, 2.8 and 2.5 Hz), 1.52 (1H, ddddd, *J* = 13.7, 13.6, 13.4, 3.2 and 3.2 Hz), 1.48 (2H, m), 1.42 (2H, m), 1.20(1H, ddd, *J* = 13.7, 13.4 and 4.1 Hz), 1.10 (1H, dd, *J* = 12.7 and 2.8 Hz), 1.05 (1H, ddd, *J* = 13.6, 13.4 and 4.1 Hz), 0.90 (3H, s), 0.84 (3H, s), 0.83 (3H, s); ^13^C NMR (CDCl_3_, 125 MHz) *δ* (ppm) 171.2, 149.3, 135.5, 135.4, 127.5, 120.7, 108.3, 68.1, 61.9, 54.6, 42.2, 40.9, 39.4, 36.6 (2C), 33.5, 23.3, 22.9, 19.1, 15.1; HR-ESI-MS *m*/*z* [M + H]^+^ 317.2086 (calcd for C_20_H_29_O_3_ 317.2117).

### Coronarin C (CZ4)

Colorless gel; ^1^H NMR (CDCl_3_, 500 MHz) *δ* (ppm) 6.83 (1H, s), 6.09 (1H, brs), 4.86 (1H, brs), 4.55 (1H, brs), 2.49 (1H, ddd, *J* = 13.3, 6.5 and 5.1 Hz), 2.40 (1H, m), 2.13 (1H, ddd, *J* = 13.3, 13.1 and 5.1 Hz), 1.97 (1H, ddd, *J* = 13.0, 12.9 and 5.1 Hz), 1.74 (4H, m), 1.63 (1H, d, *J* = 11.7 Hz), 1.58 (1H, m), 1.50 (1H, ddddd, *J* = 11.8, 4.1, 4.0, 3.8 and 3.5 Hz), 1.40 (1H, br d, *J* = 13.5 Hz), 1.32 (1H, dddd, *J* = 12.9, 12.9, 12.7 and 4.0 Hz), 1.17 (1H, ddd, *J* = 13.5, 13.3 and 4.0 Hz), 1.09 (1H, dd, *J* = 12.7 and 2.9 Hz), 1.00 (1H, ddd, *J* = 12.8, 12.8 and 4.1 Hz), 0.87 (3H, s), 0.80 (3H, s), 0.69 (3H, s); ^13^C NMR (CDCl_3_, 125 MHz) *δ* (ppm) 171.5, 148.1, 142.8, 139.3, 106.7, 96.7, 56.6, 55.7, 42.2, 39.9, 39.3, 38.4, 33.7 (2C), 24.6, 24.5, 21.9, 21.6, 19.5, 14.6; HR-ESI-MS *m*/*z* [M + H]^+^ 319.2277 (calcd for C_20_H_31_O_3_ 319.2273).

### (*E*)-14,15,16-Trinorlabda-8(17),11-dien-13-oic acid (CZ5)

Colorless gel; ^1^H NMR (CDCl_3_, 500 MHz) *δ* (ppm) 7.16 (1H, dd, *J* = 15.6 and 10.6 Hz), 5.82 (1H, d, *J* = 15.6 Hz), 4.79 (1H, d, *J* = 1.7 Hz), 4.42 (1H, d, *J* = 1.7 Hz), 2.50 (1H, d, *J* = 10.6 Hz), 2.44 (1H, ddd, *J* = 13.7, 4.5 and 2.6 Hz), 2.08 (1H, ddd, *J* = 13.7, 13.3 and 5.3 Hz), 1.71 (1H, dddd, *J* = 13.0, 5.3, 2.8 and 2.6 Hz), 1.54 (1H, ddddd, *J* = 13.7, 13.6, 13.4, 3.2 and 3.2 Hz), 1.40 (4H, m), 1.19 (1H, ddd, *J* = 13.7, 13.4 and 4.2 Hz), 1.09 (1H, dd, *J* = 13.0 and 2.8 Hz), 1.02 (1H, ddd, *J* = 13.6, 13.1 and 4.2 Hz), 0.90 (3H, s), 0.89 (3H, s), 0.84 (3H, s); ^13^C NMR (CDCl_3_, 125 MHz) *δ* (ppm) 171.4, 151.0, 148.4, 123.2, 109.0, 60.8, 54.6, 42.3, 40.9, 39.5, 36.8, 33.7 (2C), 23.4, 22.1, 19.1, 15.1; HR-ESI-MS *m*/*z* [M + H]^+^ 263.2012 (calcd for C_17_H_27_O_2_ 263.2011).

### α-Glucosidase inhibitory activity assay

The inhibitory activity of α-glucosidase was assessed using a modified method based on Kurihara *et al.*^37^ The reaction was initiated by adding 50 μL of 1.5 mM *p*-nitrophenyl-α-d-glucopyranoside and 50 μL of 0.1 U mL^−1^ α-glucosidase in 0.01 M phosphate buffer (pH 7.0) to 625 μL of sample solution. The reaction was conducted at 37 °C for 30 minutes and terminated with 0.1 M Na_2_CO_3_. Enzymatic activity was quantified by measuring absorbance at a wavelength of 401 nm. One unit of α-glucosidase activity was defined as the amount of enzyme that liberates 1.0 μM *p*-nitrophenol per minute. The IC_50_ value represented the inhibitor concentration that suppressed 50% of enzyme activity. Acarbose, a known α-glucosidase inhibitor, served as the positive control.

### ECD calculation

The conformational search was conducted using Spartan′14 (Wavefunction, Inc., Irvine, USA) with the Merck molecular force field (MMFF), followed by re-optimization at the B3LYP/6-31G* level. All conformers with a total Boltzmann distribution exceeding 95% were performed TDDFT calculation at the B3LYP/6-31G*//CAM-B3LYP/aug-cc-pVDZ level. The output files were summed to generate Boltzmann-weighted spectra using SpecDis 1.71 (Jimdo, Hamburg, Germany), with a sigma/gamma value of 0.4 eV, and these were then compared with the experimental spectra. All DFT calculations were performed using Gaussian 16 (Gaussian, Inc., Wallingford, USA).

### Molecular docking and molecular dynamics simulation

The 3D crystal of α-glucosidase from *S. cerevisiae* (PDB ID: 3A4A), was downloaded from RCSB Protein Data Bank (https://www.rcsb.org/). The 3D conformations of compounds (CZ2–CZ5) were downloaded from PubChem (https://pubchem.ncbi.nlm.nih.gov/). The 3D conformation of compound CZ1 was generated based on SMILES format by OpenBabel tool. All compounds and protein structure were prepared before performing docking by removing non-standard residues and water molecules, adding missing hydrogen atoms using the Dock Prep tool on Chimera version 1.17.3.^38^ Autodock Vina version 1.1.2 was used to perform the molecular docking interaction in this study.^39^ Molecular docking on α-glucosidase was conducted at the active site with the docking box configuration as 35.20 Å × 27.12 Å × 30.7 Å with center coordinates at *x* = 19.23, *y* = −10.14, and *z* = 24. PyMOL 3.0 software was employed to visualize 3D docking poses, while Biovia Discovery Studio 21.1 was used to illustrate 2D interactions between the enzyme and the compounds.

Molecular docking was performed to obtain the binding energies and the interactions between the α-glucosidase and compounds CZ1–CZ5. The 3D crystal structure of the α-glucosidase was obtained from the RCSB Protein Data Bank database (PDB ID: 3A4A) with non-standard residues removed and SMILES formats of natural compounds were converted to 3D structures using the OpenBabel tool. All protein and compound structures were prepared prior to docking by removing all non-standard residues, adding missing hydrogen atoms and assigning charges using the Dock Prep tool of UCSF Chimera program version 1.17.3. Next, the docking process was conducted using AutoDock Vina 1.1.2. Finally, Biovia Discovery Studio 22.1 software was used to visualize docking poses and the interaction types between α-glucosidase and ligands.

### Molecular dynamics

The MD simulations were performed on the free α-glucosidase (PDB ID: 3A4A) and the docked complexes of α-glucosidase with these five compounds using Gromacs 2024 version. The topology of α-glucosidase was prepared using CHARMS-36 force field and TIP3P GROMACS recommended water model. The topology of five compounds was prepared using CGENFF web server tool and then converted to GROMACS compatible topology file using a Python script provided by the MacKerelllab. The topology files of α-glucosidase and the compounds were manually combined using a text editor program. Next, the complex was placed in a dodecahedron box with the minimum distance of 1 nm between the solute and the box wall. All systems were solvated using the simple point charge-216 explicit water model (spc216.gro) and then neutralized using Na^+^ ions. Energy minimization was run using the steepest descent algorithm until all atomic forces in the systems were below 100 kJ mol^−1^ nm^−1^. Under position restraints, all systems were equilibrated in two stages with a time step of 2 fs and the duration of 1 ns. In the first stage, all systems were equilibrated in an NVT ensemble using the V-rescale thermostat at the temperature of 300 K. The second equilibration stage was conducted in an NPT ensemble using the C-rescale barostat at the pressure of 1 bar. To account for electrostatic forces, the Ewald Particle Mesh (PME) method was used. A 1 nm cutoff was applied to treat short-range electrostatics and van der Waals interactions. Hydrogen bonds were constrained in both equilibration and production steps using the LINCS algorithm. Finally, the production run was conducted for 100 ns with a trajectory snapshot saved every 10 ps.

### *In silico* ADME profiles

*In silico* ADME predictions for the compounds were performed using the SwissADME web tool (www.swissadme.ch, accessed 27 December 2024).^40^ The SMILES format of each compound was used to compute key physicochemical properties and evaluated for their drug-likeness. The compounds were also analysed for their pharmacokinetic profiles, including gastrointestinal absorption and interaction with cytochrome P450. The *in silico* toxicity profiles of the compounds were analyzed using the DEEP-PK web tool (https://biosig.lab.uq.edu.au/deeppk/, accessed 27 December 2024).^35^ Toxicity predictions included classification into toxicity categories and estimation of the median lethal dose (LD_50_) and interaction between compounds and biological targets critical to key physiological processes, offering valuable insights into the safety and potential risks of these compounds.

## Author contributions

Conceptualization: T. H. L; isolation and identification: D. N. T. L, T. H. L, P. H. D, and H. X. N.; bioassay: T. H. L, T. N. V. D and T. Q. T.; computational studies: M. H. N. and T. H. N. L.; writing – original draft preparation: T. H. L., P. H. D., and M. H. N.; writing – review and editing: T. H. L. and M. H. N.; supervision: M.T.T.N; project administration: M. T. T. N. All authors have read and agreed to the published version of the manuscript.

## Conflicts of interest

The authors declare no competing financial interest.

## Supplementary Material

RA-015-D5RA03418C-s001

## Data Availability

The datasets supporting this article have been uploaded as part of the ESI.†

## References

[cit1] Joshi S. R., Standl E., Tong N., Shah P., Kalra S., Rathod R. (2015). Expet Opin. Pharmacother..

[cit2] Holman R. R., Cull C. A., Turner R. C. (1999). Diabetes Care.

[cit3] Khwaja N. U., Arunagirinathan G. (2021). Curr. Drug Saf..

[cit4] Tannous S., Stellbrinck T., Hoter A., Naim H. Y. (2023). Front. Mol. Biosci..

[cit5] Loizzo M. R. , Bonesi M., Nabavi S. M., Sobarzo-Sánchez E., Rastrelli L. and Tundis R., NP Targeting Clin Rel Enzymes, 2017, pp. 135–161

[cit6] Bisio A., De Mieri M., Milella L., Schito A. M., Parricchi A., Russo D., Alfei S., Lapillo M., Tuccinardi T., Hamburger M. (2017). J. Nat. Prod..

[cit7] Hinkson I. V., Madej B., Stahlberg E. A. (2020). Front. Pharmacol.

[cit8] Sun D., Gao W., Hu H., Zhou S. (2022). Acta Pharm. Sin. B.

[cit9] Prajapati R. N., Bhushan B., Singh K., Chopra H., Kumar S., Agrawal M., Pathak D., Chanchal D. K., Laxmikant (2024). Curr. Pharm. Biotechnol..

[cit10] Durán-Iturbide N. A., Díaz-Eufracio B. I., Medina-Franco J. L. (2020). ACS Omega.

[cit11] Paul Gleeson M., Hersey A., Hannongbua S. (2011). Curr. Top. Med. Chem..

[cit12] Guo Y., Han Z., Zhang J., Lu Y., Li C., Liu G. (2024). Heliyon.

[cit13] Kumar A., Singh S. K., Singh V. K., Kant C., Singh A. K., Tripathi V., Singh K., Sharma V. K., Singh J. (2022). J. S. Afr. Bot..

[cit14] Do T. L. , Vietnamese Traditional Medicinal Plants and Drugs, Publishing House of Medicine, Hanoi, 2001

[cit15] D. H. Bich , D. Q. Chung, B. X. Chuong, N. T. Dong, D. T. Dam, P. V. Hien and T. Toan, ed. The Medicinal Plants and Animals in Vietnam, Hanoi Science and Technology, Publishing House, Hanoi, 2004

[cit16] Lee T. K., Trinh T. A., Lee S. R., Kim S., So H. M., Moon E., Hwang G. S., Kang K. S., Kim J. H., Yamabe N. (2019). Bioorg. Chem..

[cit17] Chen I.-N., Chang C.-C., Ng C.-C., Wang C.-Y., Shyu Y.-T., Chang T.-L. (2008). Plant Foods Hum. Nutr..

[cit18] Ahmed Hamdi O. A., Syed Abdul Rahman S. N., Awang K., Abdul Wahab N., Looi C. Y., Thomas N. F., Abd Malek S. N. (2014). Sci. World J..

[cit19] Hamdi O. A. A., Ye L. J., Kamarudin M. N. A., Hazni H., Paydar M., Looi C. Y., Shilpi J. A., Kadir H. A., Awang K. (2015). Rec. Nat. Prod..

[cit20] Tran Q. T., Wong W. F., Chai C. L. (2017). Pharmacol. Res..

[cit21] Saha P., Rahman F. I., Hussain F., Rahman S. A., Rahman M. M. (2022). Front. Pharmacol.

[cit22] Chinou I. (2005). Curr. Med. Chem..

[cit23] Wu J., Zhang W., Xu J., Wang Y., He X. (2025). Chem. Biodiversity.

[cit24] Xu H.-X., Hui D., Sim K.-Y. (1995). Nat. Prod. Lett..

[cit25] Itokawa H., Morita H., Katou I., Takeya K., Cavalheiro A. J., de Oliveira R. C., Ishige M., Motidome M. (1988). Planta Med..

[cit26] Akiyama K., Kikuzaki H., Aoki T., Okuda A., Lajis N. H., Nakatani N. (2006). J. Nat. Prod..

[cit27] Rosak C., Mertes G. (2012). Diabetes, Metab. Syndr. Obes.: Targets Ther..

[cit28] Gafar M. K., Salim F., Shah S. A. A., Azman I. I. N., Ahmad R. (2023). Phytochem. Lett..

[cit29] Lu C.-L., Wang L.-N., Li Y.-J., Fan Q.-F., Huang Q.-H., Chen J.-J. (2022). Nat. Prod. Res..

[cit30] Fan Z. Y., Pang W., Yu Y. Y., Xu S. H., Cheng L. P. (2025). Bioorg. Chem..

[cit31] Viigand K., Visnapuu T., Mardo K., Aasamets A., Alamäe T. (2016). Yeast.

[cit32] Sakulkeo O., Wattanapiromsakul C., Pitakbut T., Dej-Adisai S. (2022). Molecules.

[cit33] Amin S., Ullah B., Ali M., Rauf A., Khan H., Uriarte E., Sobarzo-Sánchez E. (2019). Molecules.

[cit34] Yamamoto K., Miyake H., Kusunoki M., Osaki S. (2010). FEBS J..

[cit35] Myung Y., de Sá A. G., Ascher D. B. (2024). Nucleic Acids Res..

[cit36] Cavasotto C. N., Scardino V. (2022). ACS Omega.

[cit37] Kurihara H., Ando J., Hatano M., Kawabata J. (1995). Bioorg. Med. Chem. Lett..

[cit38] Pettersen E. F., Goddard T. D., Huang C. C., Couch G. S., Greenblatt D. M., Meng E. C., Ferrin T. E. (2004). J. Comput. Chem..

[cit39] Trott O., Olson A. J. (2010). J. Comput. Chem..

[cit40] Daina A., Michielin O., Zoete V. (2017). Sci. Rep..

