# Supplementary material for: Isolation, structural elucidation, and integrated biological and computational evaluation of antidiabetic labdane diterpenes from Curcuma zedoaria rhizomes
Source: RSC Adv. 2025 Jun 26;15(27):21879–89. doi: 10.1039/d5ra03418c (PMC12199309; doi:10.1039/d5ra03418c)
Supplement: RA-015-D5RA03418C-s001 [file RA-015-D5RA03418C-s001.pdf]

This journal is © The Royal Society of Chemistry 2025

SUPPORTING INFORMATION

Figure S1. ^{1}H NMR spectrum of compound **CZ1** (500 MHz, $CDCl_3$).. S3
Figure S2. ^{13}C NMR spectrum of compound **CZ1** (125 MHz, $CDCl_3$).. S3
Figure S3. HSQC spectrum of compound **CZ1**.. S4
Figure S4. HMBC spectrum of compound **CZ1**.. S4
Figure S5. NOESY spectrum of compound **CZ1**.. S5
Figure S6. IR spectrum of compound **CZ1**.. S5
Figure S7. MS spectrum of compound **CZ1**.. S6
Figure S8. ^{1}H NMR spectrum of compound **CZ2** (500 MHz, $CDCl_3$).. S6
Figure S9. ^{13}C NMR spectrum of compound **CZ2** (125 MHz, $CDCl_3$).. S7
Figure S10. MS spectrum of compound **CZ2**.. S7
Figure S11. ^{1}H NMR spectrum of compound **CZ3** (500 MHz, $CDCl_3$).. S8
Figure S12. ^{13}C NMR spectrum of compound **CZ3** (125 MHz, $CDCl_3$).. S8
Figure S13. MS spectrum of compound **CZ3**.. S9
Figure S14. ^{1}H NMR spectrum of compound **CZ4** (500 MHz, $CDCl_3$).. S9
Figure S15. ^{13}C NMR spectrum of compound **CZ4** (125 MHz, $CDCl_3$).. S10
Figure S16. MS spectrum of compound **CZ4**.. S10
Figure S17. ^{1}H NMR spectrum of compound **CZ5** (500 MHz, $CDCl_3$).. S11
Figure S18. ^{13}C NMR spectrum of compound **CZ5** (125 MHz, $CDCl_3$).. S11
Figure S19. MS spectrum of compound **CZ5**.. S12

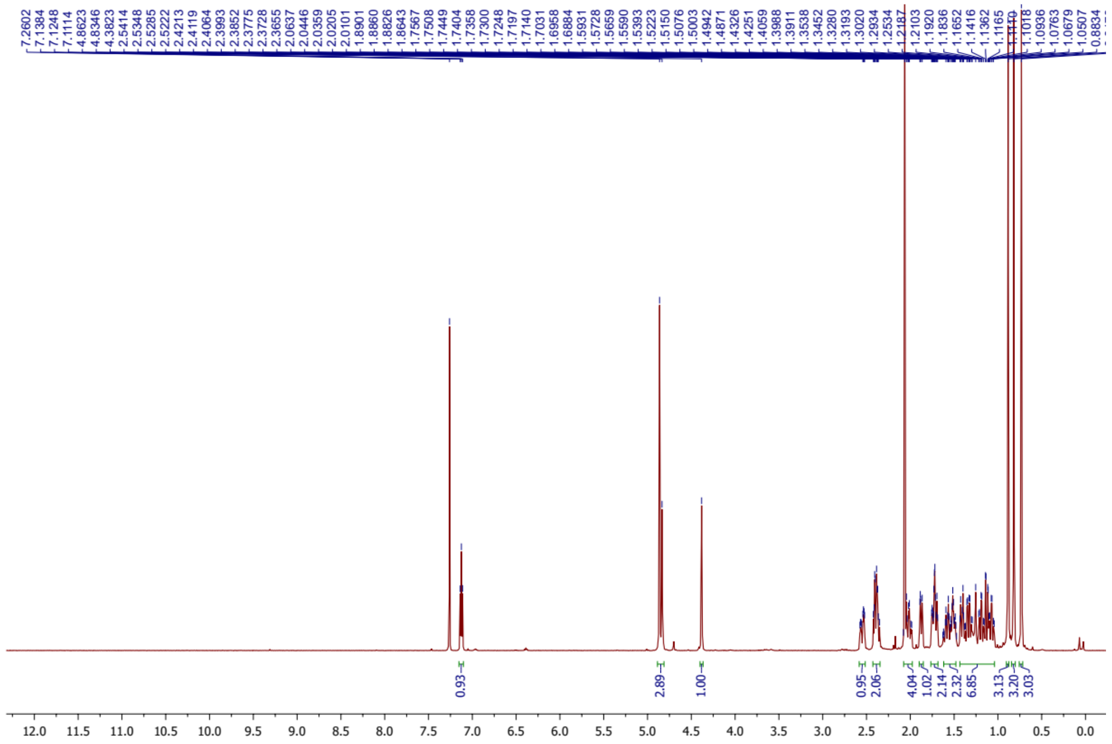

Figure S1. ^{1}H NMR spectrum of compound **CZ1** (500 MHz, $CDCl_3$)

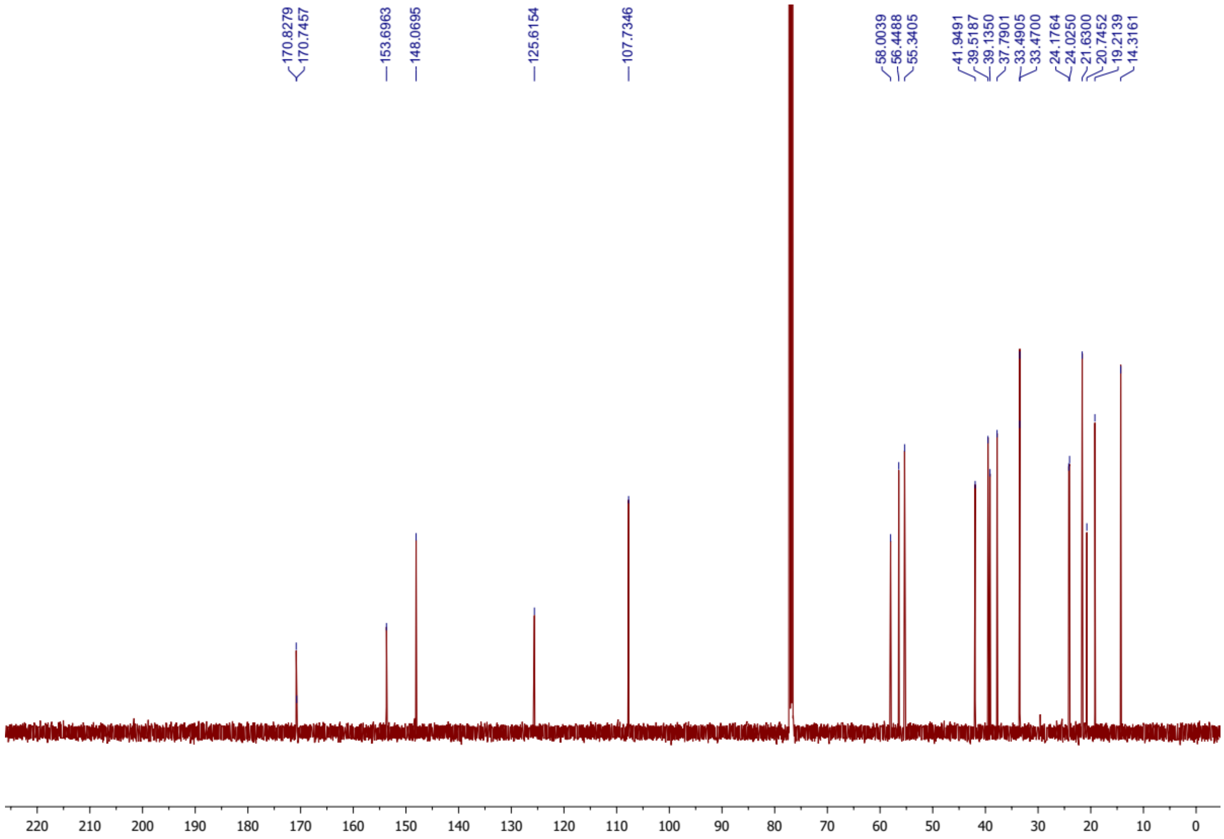

Figure S2. ^{13}C NMR spectrum of compound **CZ1** (125 MHz, $CDCl_3$)

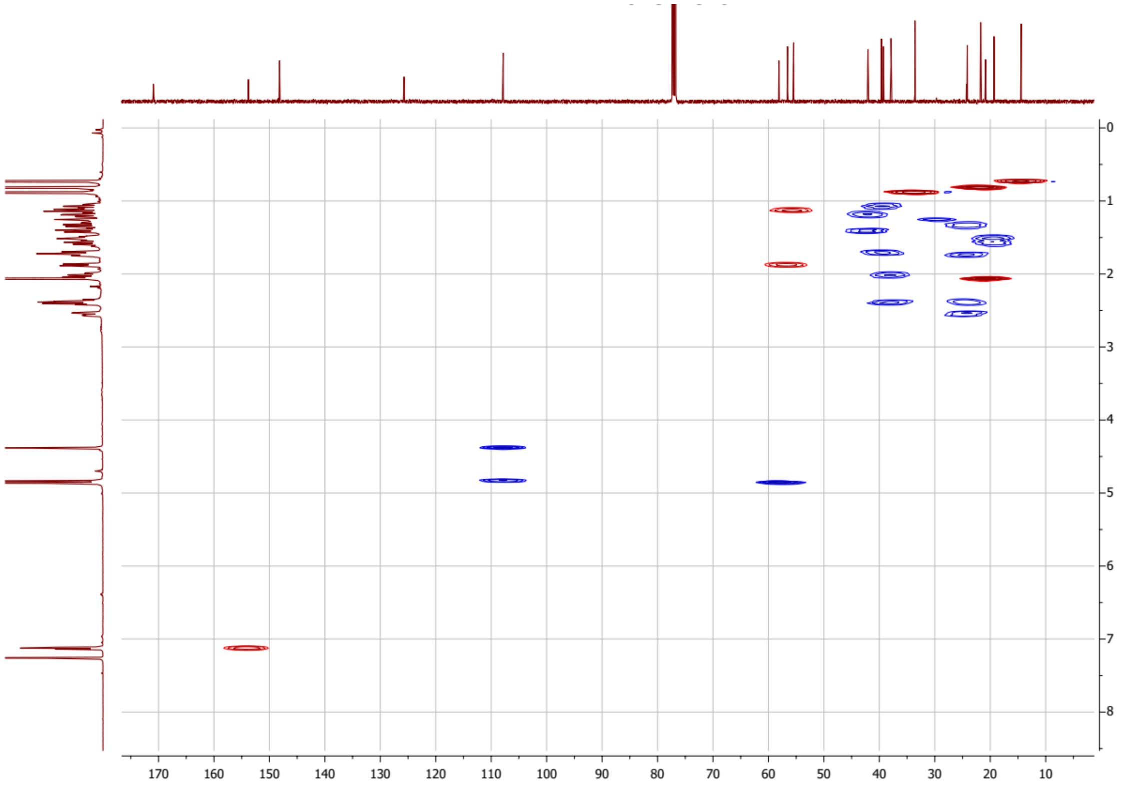

Figure S3. HSQC spectrum of compound **CZ1**

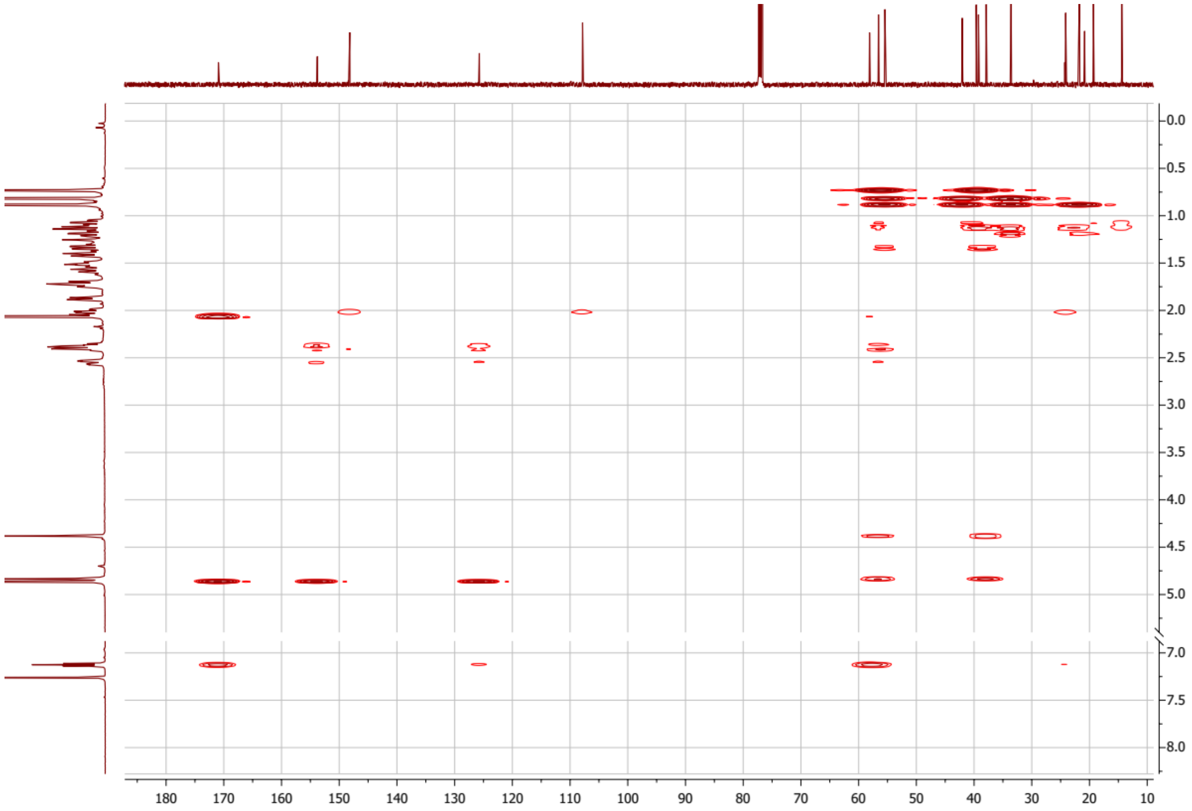

Figure S4. HMBC spectrum of compound **CZ1**

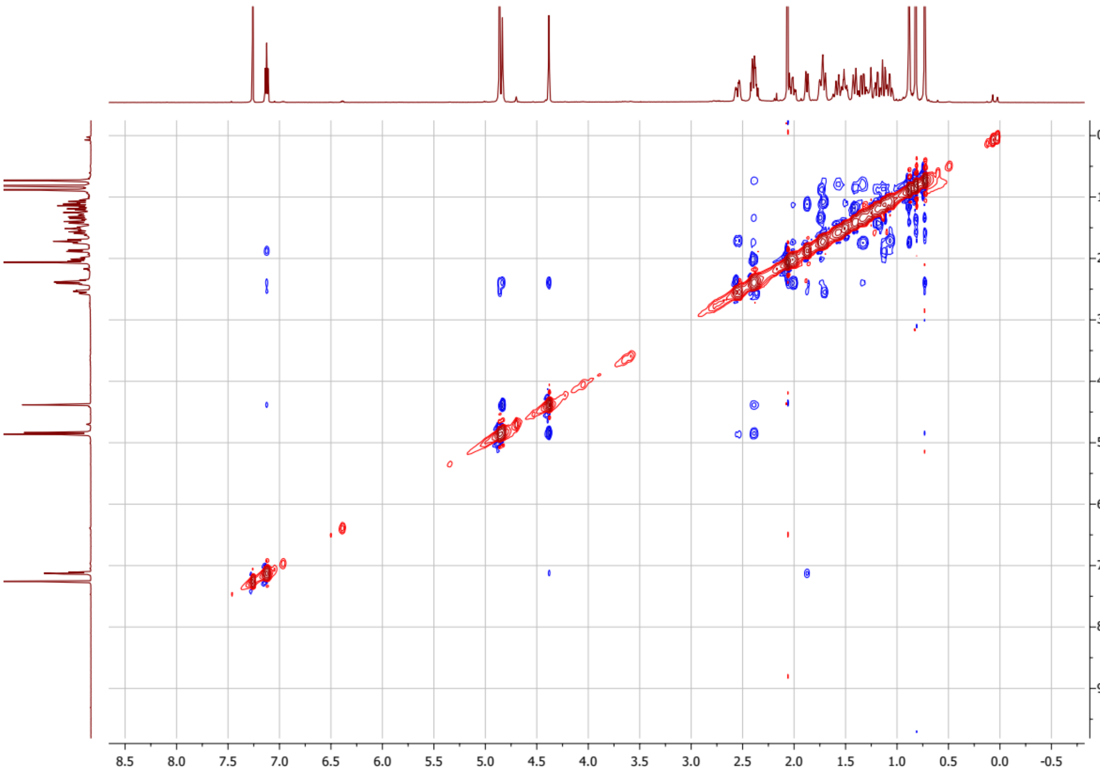

Figure S5. NOESY spectrum of compound **CZ1**

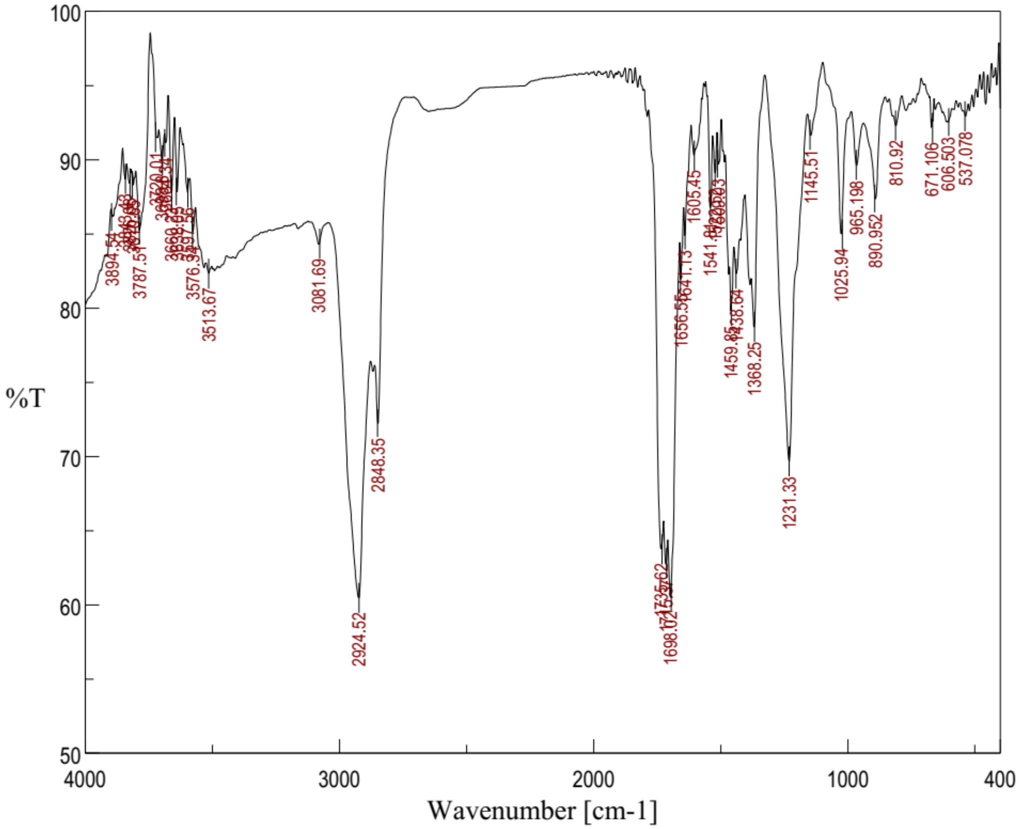

Figure S6. IR spectrum of compound **CZ1**

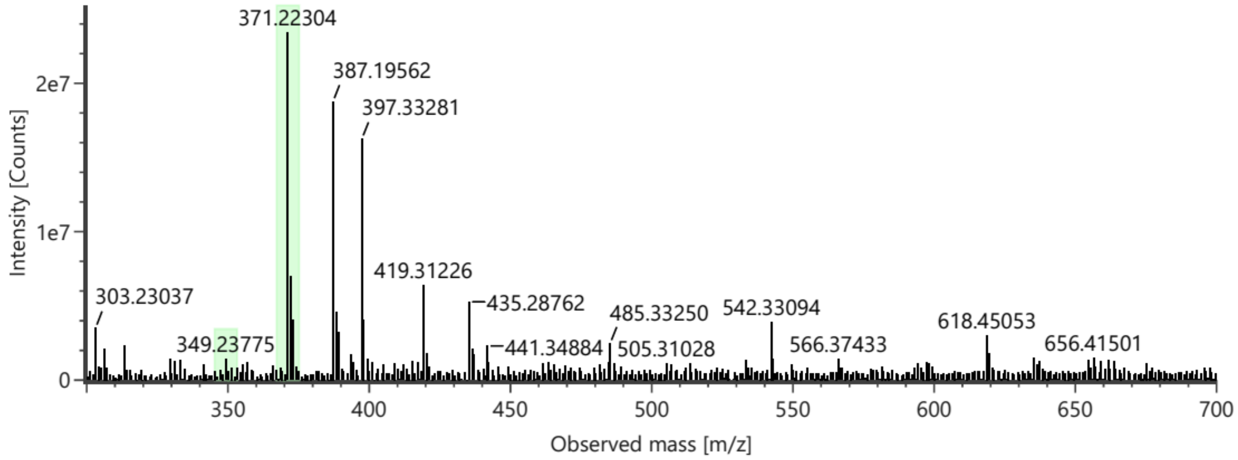

Figure S7. MS spectrum of compound **CZ1**

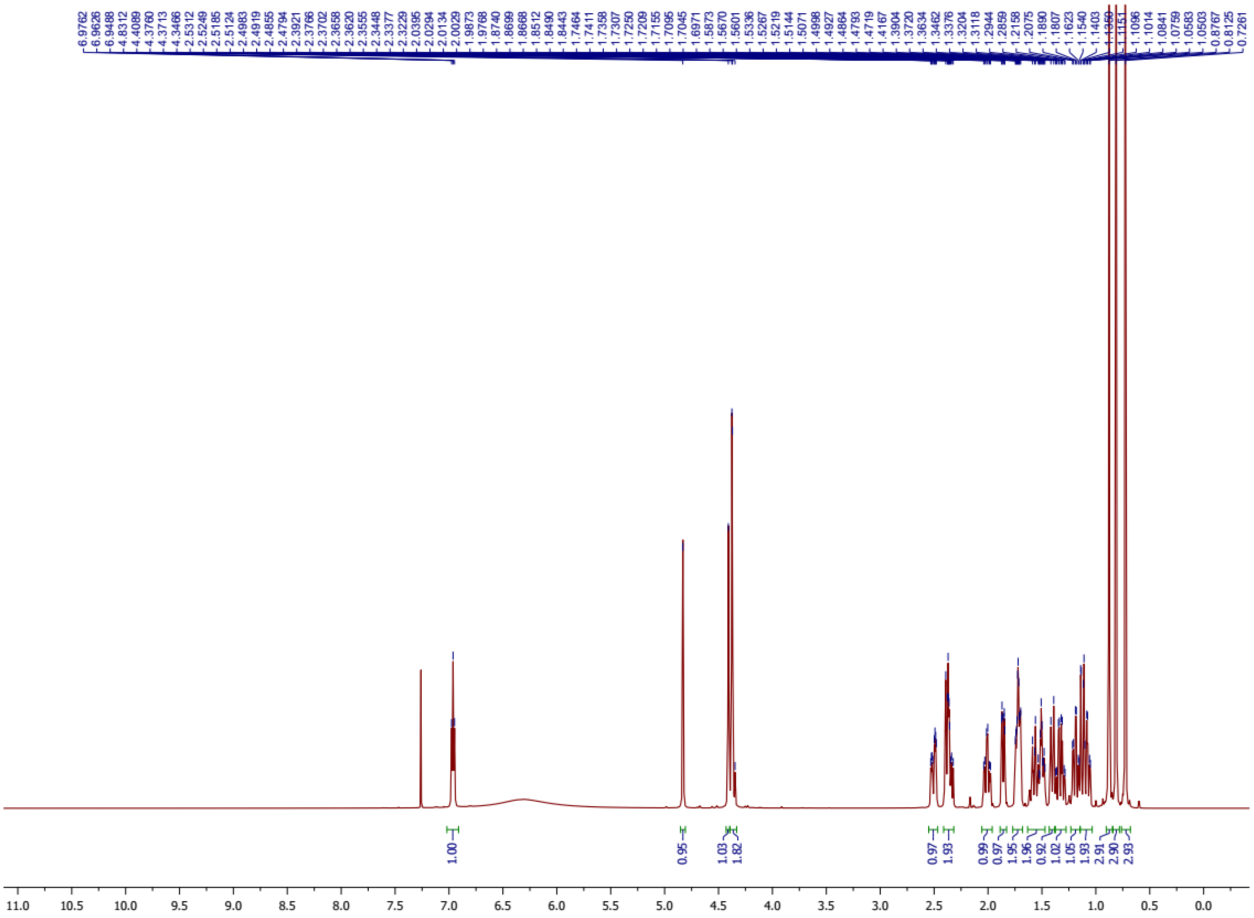

Figure S8. ^{1}H NMR spectrum of compound **CZ2** (500 MHz, $CDCl_3$)

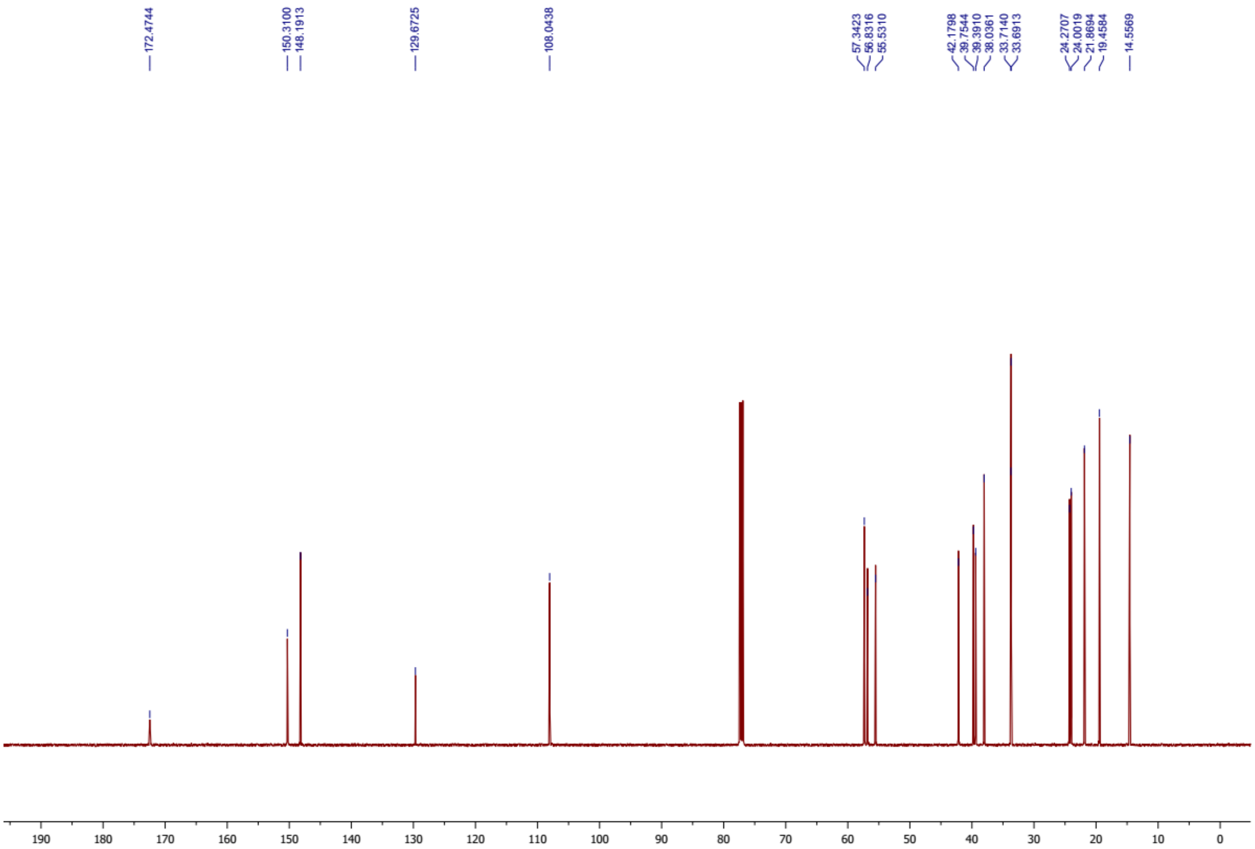

Figure S9. ^{13}C NMR spectrum of compound **CZ2** (125 MHz, $CDCl_3$)

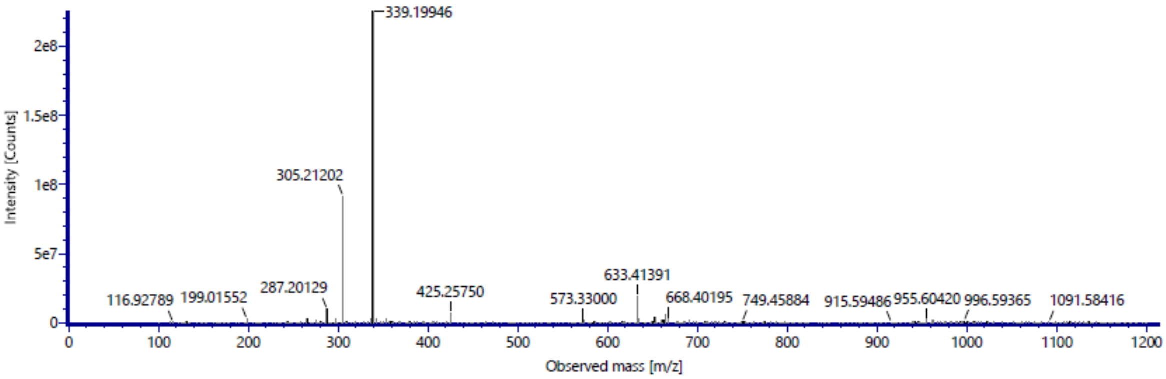

Figure S10. MS spectrum of compound **CZ2**

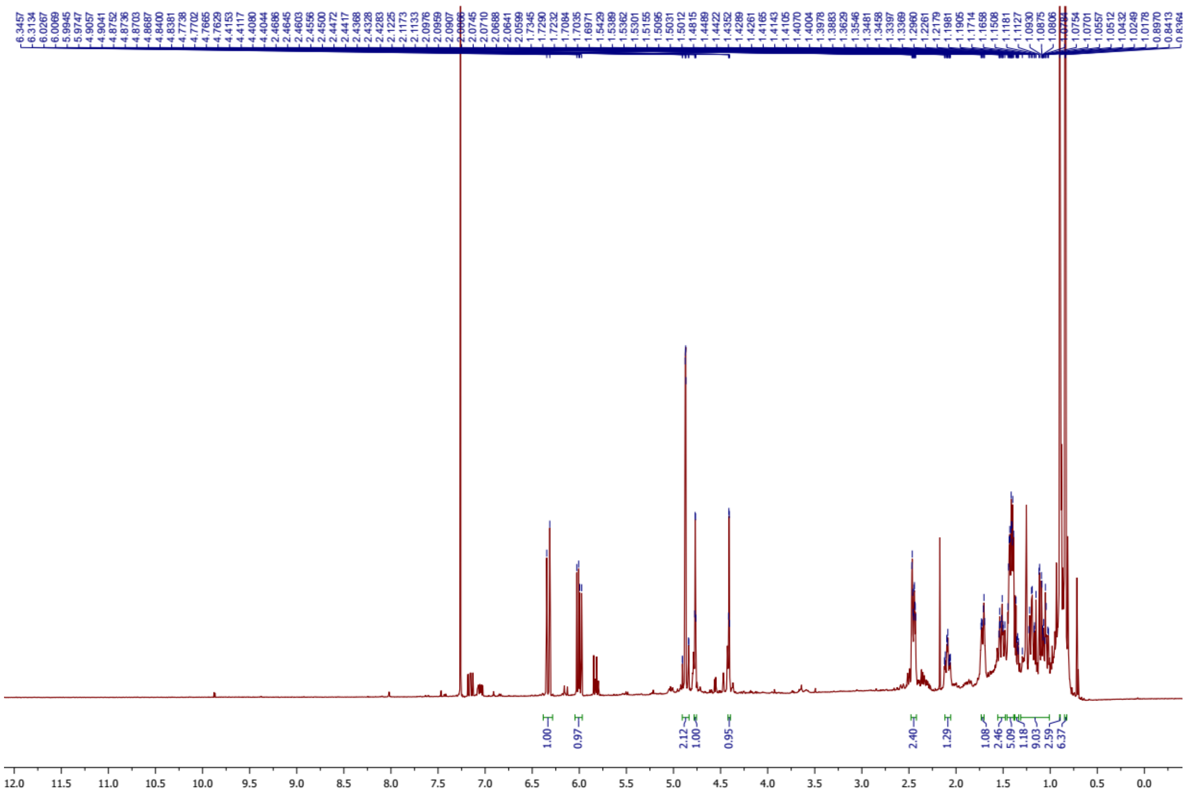

Figure S11. ^{1}H NMR spectrum of compound **CZ3** (500 MHz, $CDCl_3$)

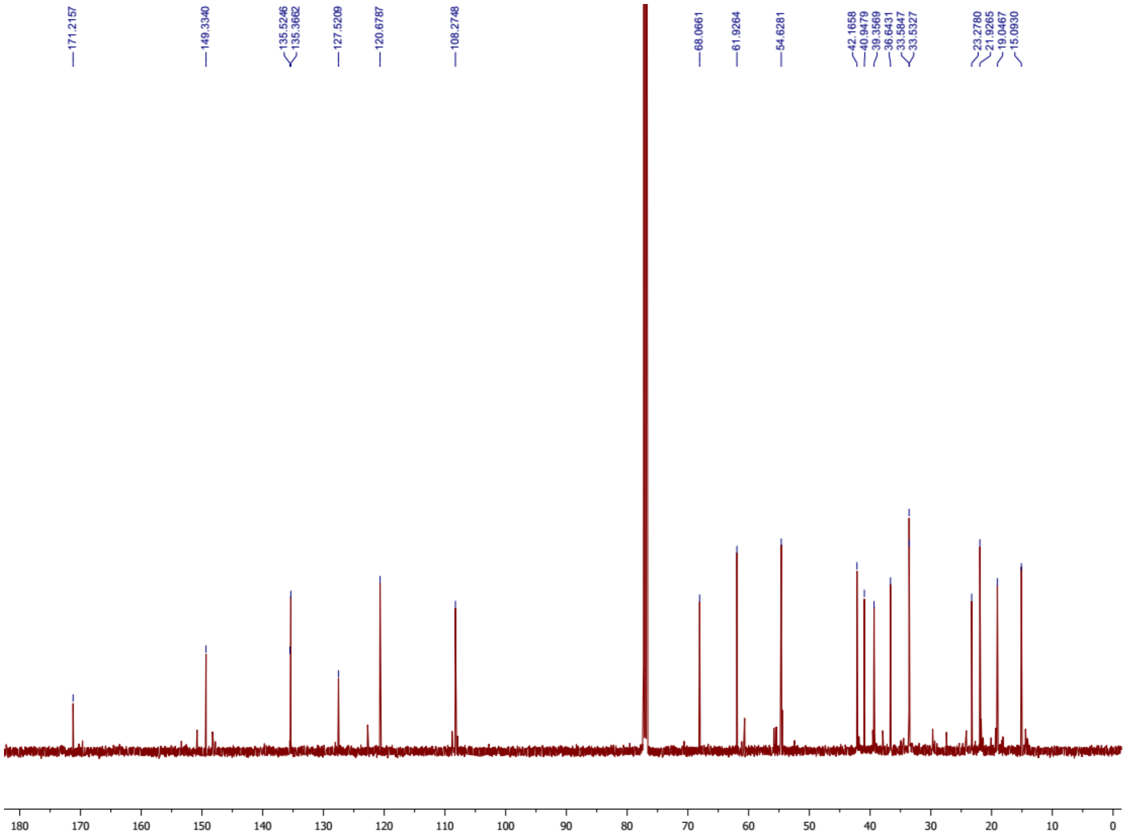

Figure S12. ^{13}C NMR spectrum of compound **CZ3** (125 MHz, $CDCl_3$)

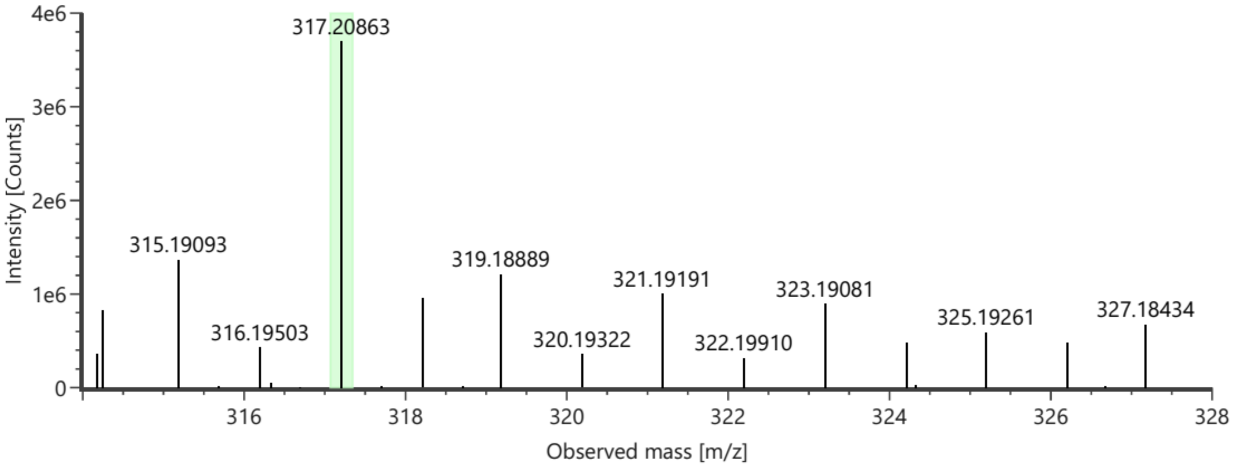

Figure S13. MS spectrum of compound **CZ3**

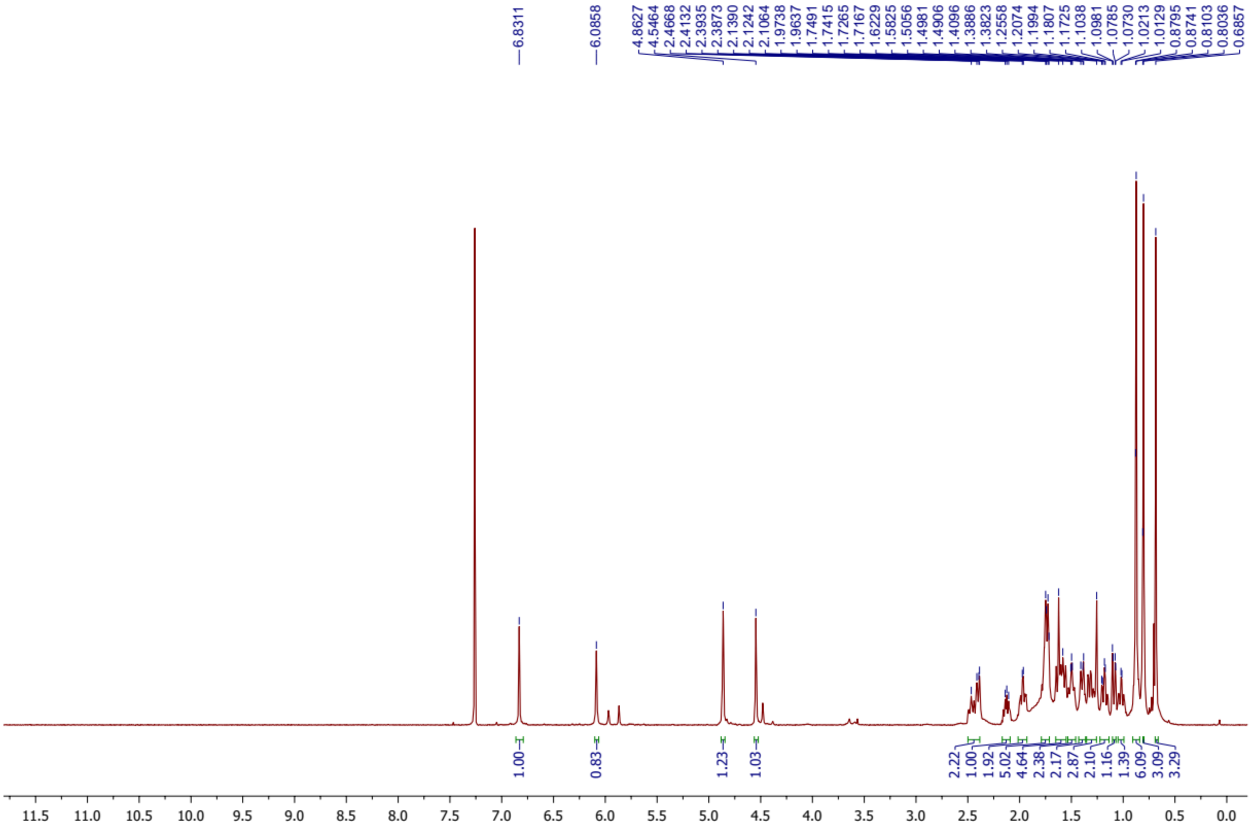

Figure S14. ^{1}H NMR spectrum of compound **CZ4** (500 MHz, $CDCl_3$)

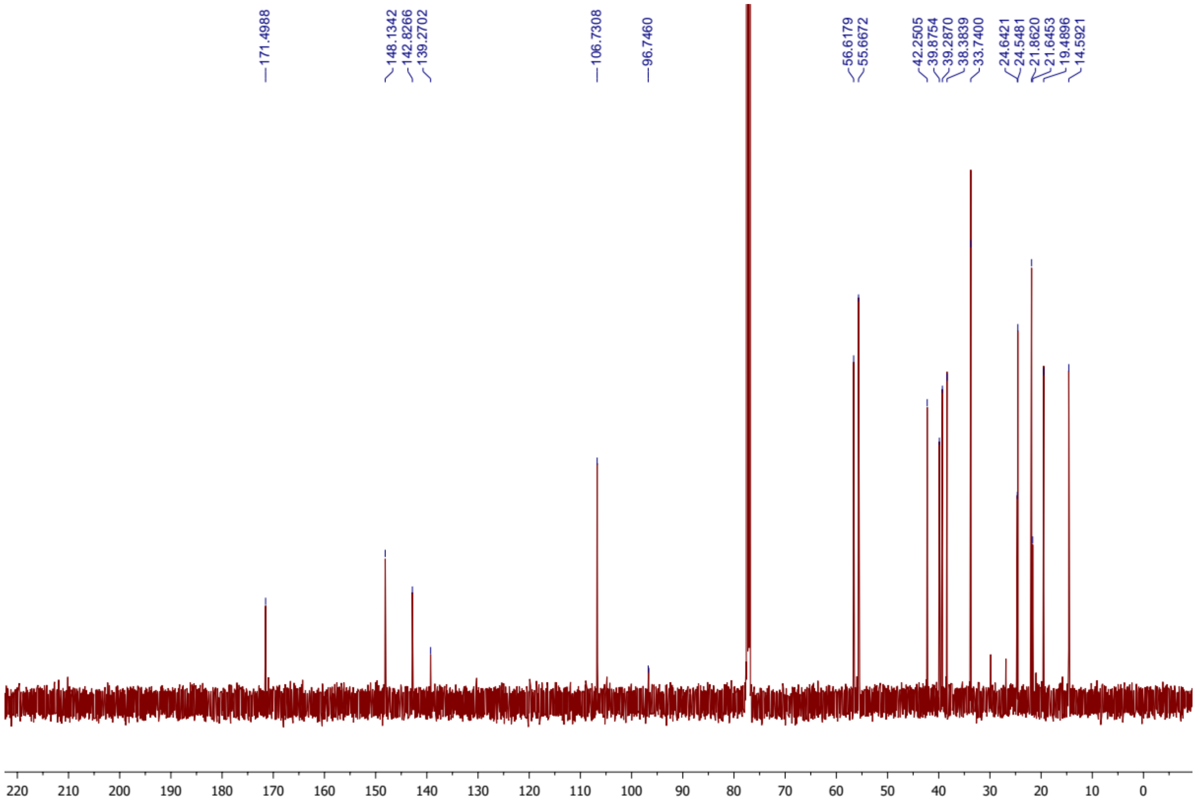

Figure S15. ^{13}C NMR spectrum of compound **CZ4** (125 MHz, $CDCl_3$)

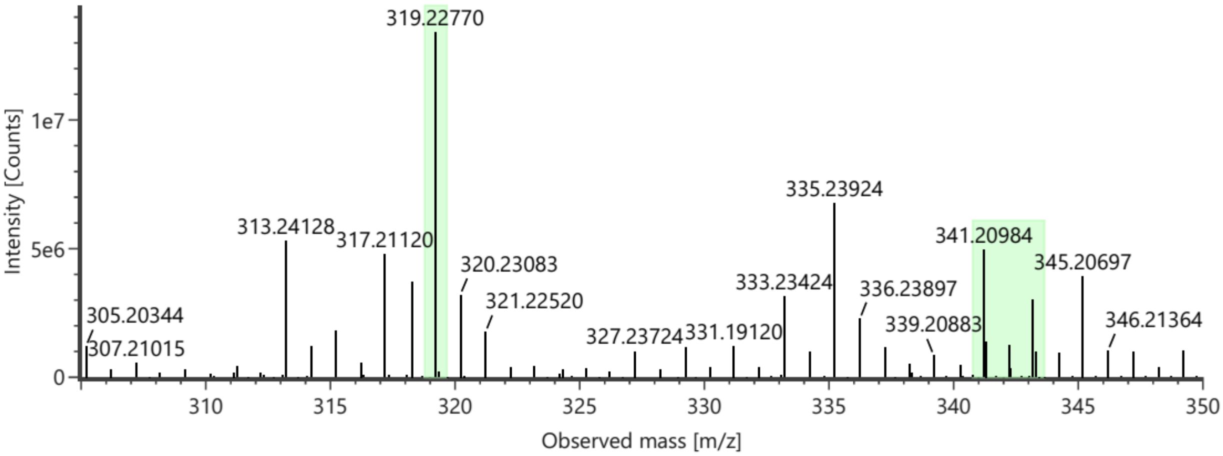

Figure S16. MS spectrum of compound **CZ4**

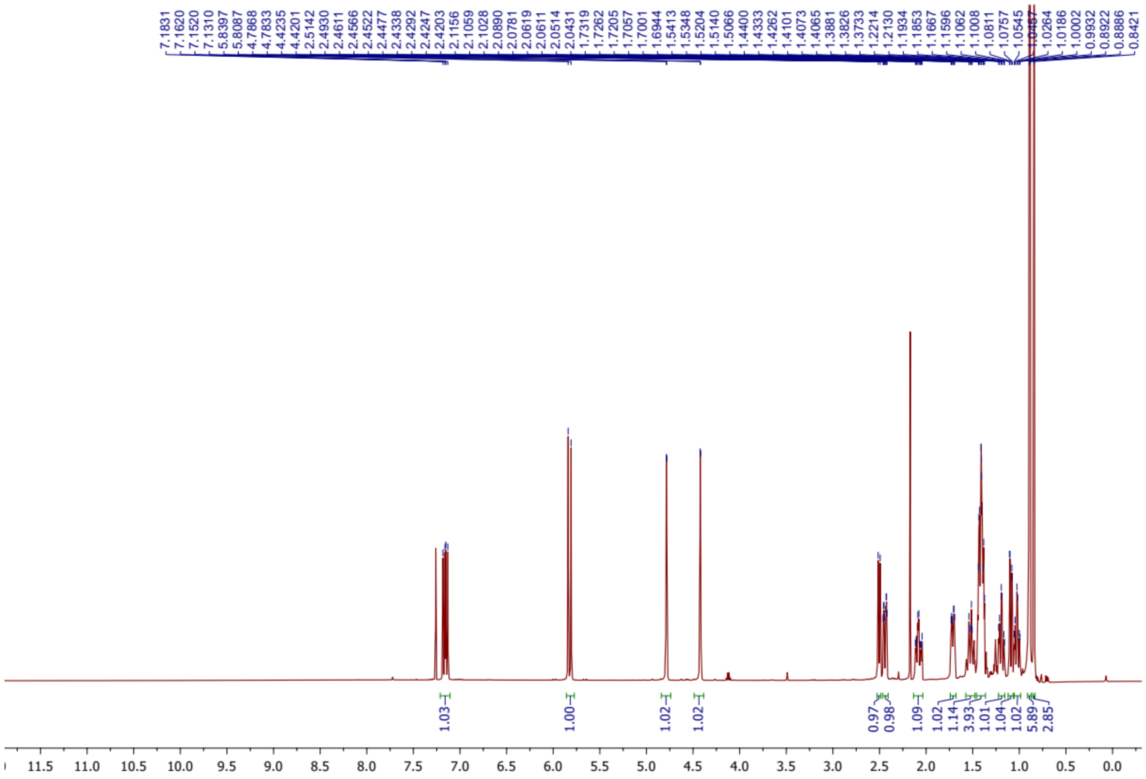

Figure S17. ^{1}H NMR spectrum of compound **CZ5** (500 MHz, $CDCl_3$)

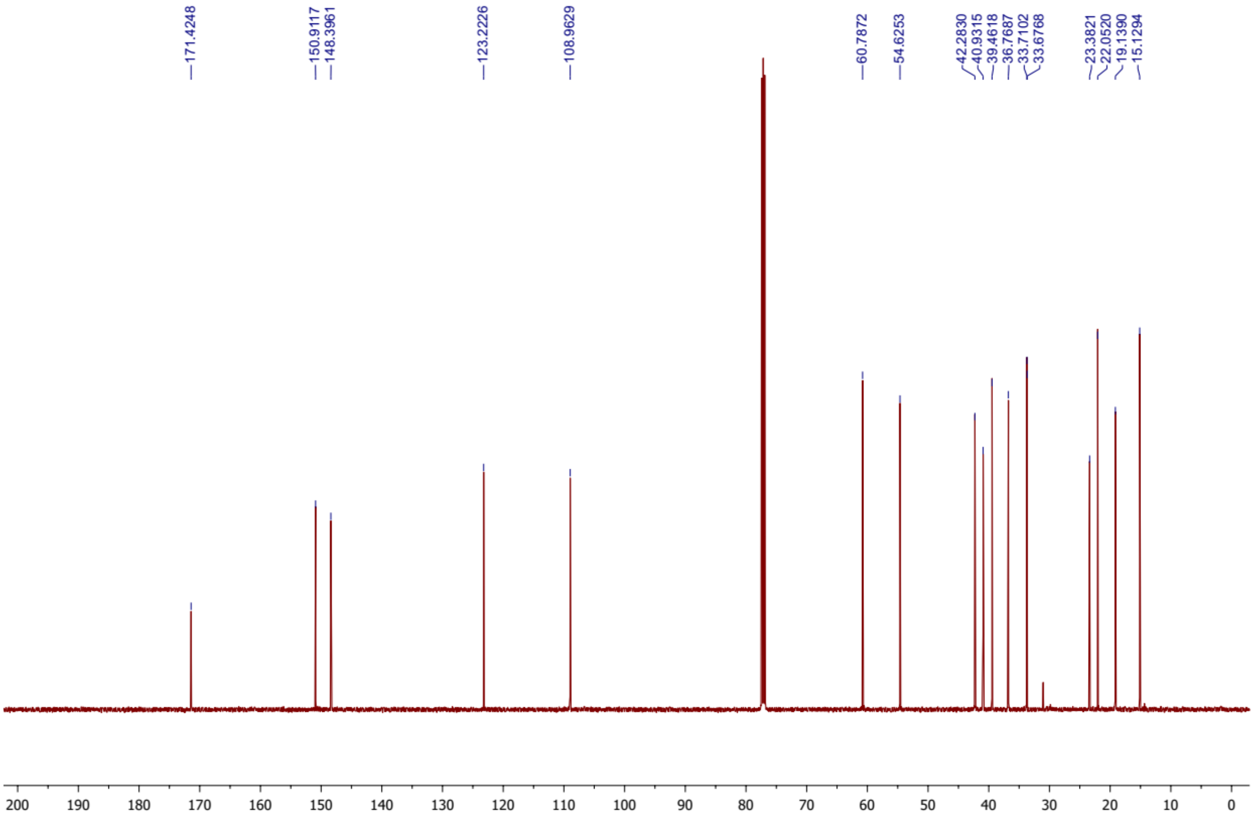

Figure S18. ^{13}C NMR spectrum of compound **CZ5** (125 MHz, $CDCl_3$)

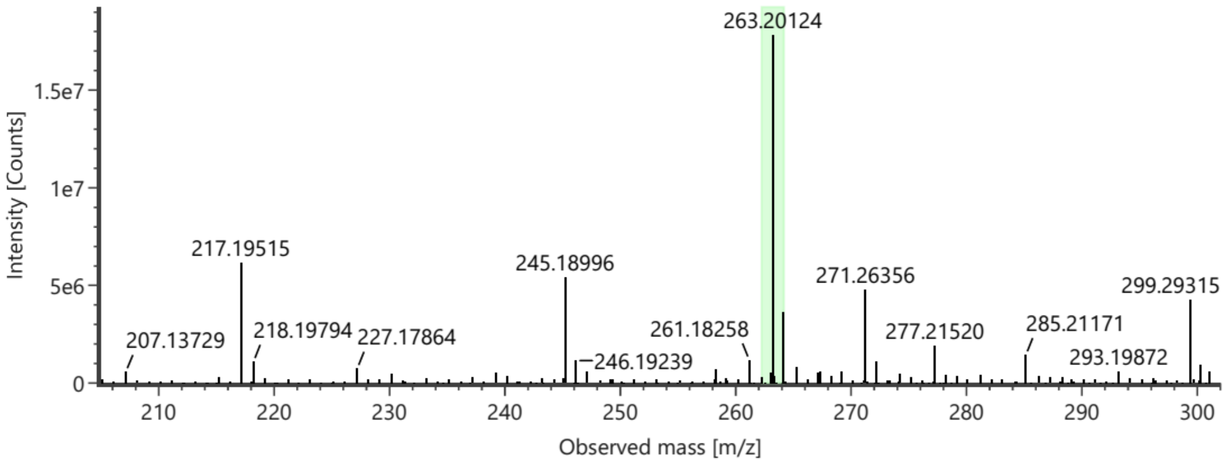

Figure S19. MS spectrum of compound **CZ5**
